# Anticancer bispecific antibody R&D advances: a study focusing on research trend worldwide and in China

**DOI:** 10.1186/s13045-021-01126-x

**Published:** 2021-08-16

**Authors:** Zhonghan Zhang, Fan Luo, Jiaxin Cao, Feiteng Lu, Yang Zhang, Yuxiang Ma, Kangmei Zeng, Li Zhang, Hongyun Zhao

**Affiliations:** 1 Department of Medical Oncology, Sun Yat-Sen University Cancer Center, State Key Laboratory of Oncology in South China, Collaborative Innovation Center for Cancer Medicine, 651 Dongfeng Road East, Guangzhou, Guangdong 510060 People’s Republic of China; 2 Department of Clinical Research, Sun Yat-Sen University Cancer Center, State Key Laboratory of Oncology in South China, Collaborative Innovation Center for Cancer Medicine, Guangzhou, People’s Republic of China

**Keywords:** Bispecific antibody, BsAbs, China, Worldwide, Clinical trials

## Abstract

**Background:**

The bispecific antibody (bsAbs) research around the world has undergone great changes. We analyzed the global trend of bsAbs research and compared the differences in clinical research of bsAbs between China and worldwide.

**Methods:**

BsAbs research clinical trials information was retrieved through the online open-resource clinical trial registration platform. Research information including organizations, identity numbers, locations, phases, participating centers, conditions, status, enrollment, targets, spectrums of mechanism of action (MOA), and start date was collected. Clinical trials were divided into two categories based on the attributes of pharmaceutical companies (international or China-initiated or involved).

**Results:**

From 1997 to 2020, 272 clinical trials regarding bsAbs research were retrieved. Twenty-nine percent of the studies were contributed by companies from Chinese institutions, which followed the USA and ranked second. The clinical trials of bsAbs are mainly concentrated on phase I (*n* = 161), phase I/II (*n* = 54), and phase II (*n* = 51), and the number of phase III trials is still rare (*n* = 4). Tumor species distribution analysis shows that there are significantly higher focuses on gastric cancer (*n* = 18), esophageal/gastroesophageal junction cancer (*n* = 16), bladder cancer (*n* = 10), biliary malignant tumor (*n* = 8), nasopharyngeal cancer (*n* = 6), and thymic cancer (*n* = 2) in China. BsAbs target and spectrums of MOA analysis showed that international companies mainly focus on bsAbs with CD3-based (*n* = 63) target with MOA of T-cell redirection, while researches in China pay more attention to PD-1 (*n* = 9)/PD-L1 (*n* = 7) axises with MOA of double immune checkpoint blocking.

**Conclusion:**

Global bsAbs research increased rapidly during the 1997 to 2020 period. The developed countries in America and Europe are leading the trend of bsAbs research. Anticancer bsAbs clinical research in China is booming and chasing after the world trend.

**Supplementary Information:**

The online version contains supplementary material available at 10.1186/s13045-021-01126-x.

**To The Editor**

BsAb anti-tumor clinical researches have continued to grow rapidly worldwide and in China [1–4], whereas scientific research and development are still unbalanced [5–7]. This study helps to comprehensively understand the global development of bsAbs and the trend of technological innovation development in China with this aspect.

## Booming development in anticancer bispecific antibodies investigation

**Fig. 1:**
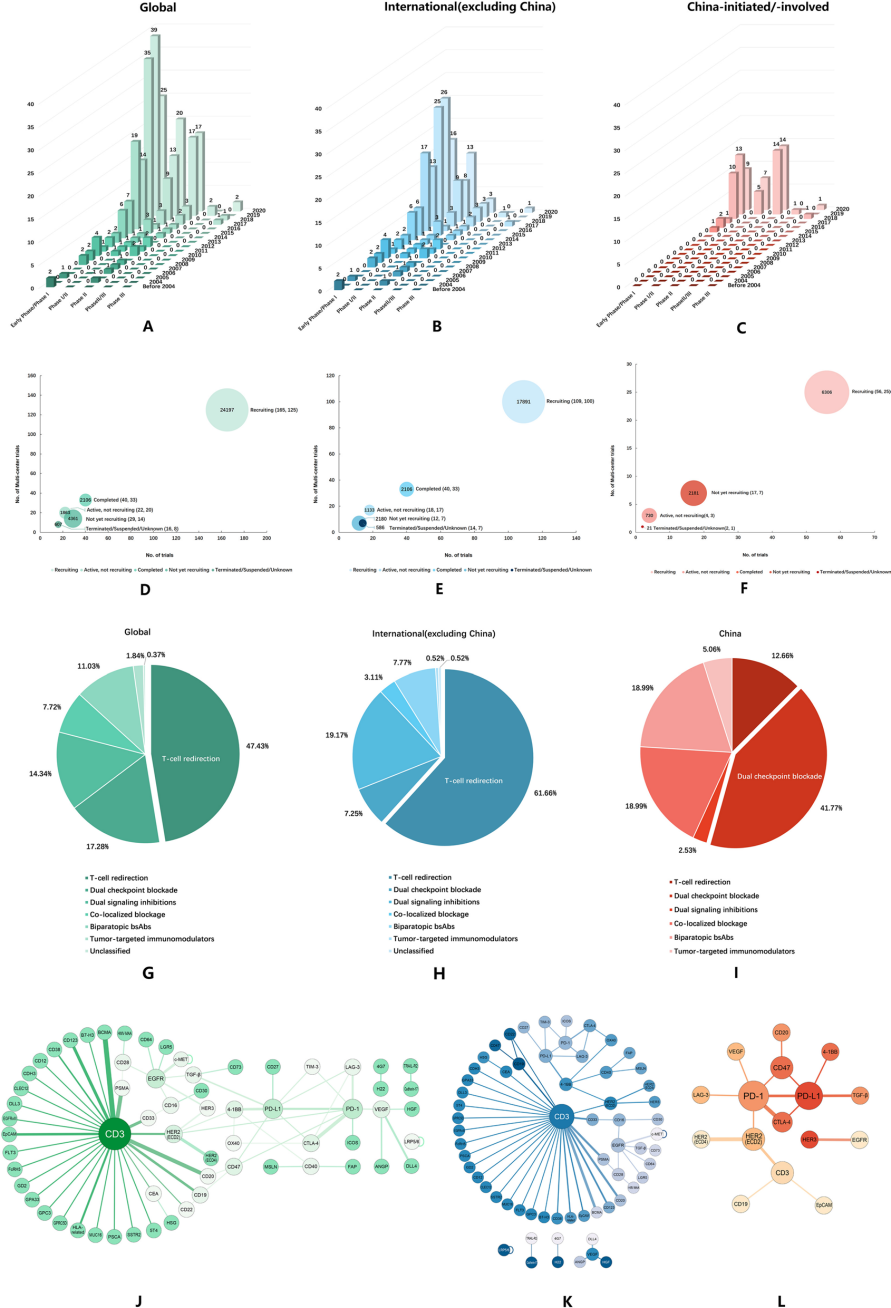
The trend of anticancer bsAbs clinical trials with different clinical stages and status (up to December 2020). **a** Clinical stages of overall clinical trials. **b** Clinical stages of international companies. **c** Clinical stages of China-initiated or involved companies. **d** Research status and enrollment of overall clinical trials. **e** Research status and enrollment of international companies. **f** Research status and enrollment of China-initiated or involved companies. The MOA of bsAbs clinical trials. **g** MOA of overall clinical trials. **h** MOA of international companies. **i** MOA of China-initiated or involved companies. Network graph characterizing the target pairs of bispecific antibodies. Each dot in the figure is a target, and each line connecting two circular nodes represents a bsAbs. The size of the dot is proportional to the number of pairs of the target with other different targets, and the width of the line is proportional to the number of clinical trials of the target. **j** Target pairs of overall clinical trials. **k** Target pairs of international companies. **l** Target pairs of China-initiated or involved companies

International R&D pharmaceutical enterprises have conducted 193 bsAbs clinical trials (Additional file 1: Table S1), and China-initiated or involved R&D pharmaceutical enterprises have conducted 79 clinical trials (Additional file 1: Table S2). The characteristics of bsAbs research at domestic and international pharmaceutical companies are different in many aspects (Additional file 1: Table S3). Overall distribution of bsAbs clinical research is illustrated in a topographic map (Additional file 2: Fig. S1). A total of 98 locations in China participate in anticancer bsAbs research, making it the second highest country to USA (Fig. 1a; Additional file 1: Table S4). Beijing, Guangdong, Henan, Taiwan, Shandong, Zhejiang, and Shanghai are the main research areas of bsAbs clinical research in China (Additional file 2: Fig. S1B and Additional file 1: Table S5).

## More expansion of early phase studies and study scale

Global bsAbs clinical trials are mainly concentrated on phase I (*n* = 161), phase I/II, and phase II, and phase III trials are still rare (Fig. 1a). International pharmaceutical companies’ anti-tumor bsAbs research has entered a stage of rapid development after 2014 (Fig. 1b), while China outbroke since 2017 (Fig. 1c). The number of phase I/II and II/III research designs has gradually increased, and new bsAbs research designs have emerged. Most of the clinical studies are under recruitment, and a few clinical studies have been suspended for various reasons (Fig. 1d–f).

## The mechanism of action of bispecific antibodies clinical trials

The mechanism of action (MOA) of bsAbs included six categories (Additional file 1: Table S6). The global and international companies MOA is mainly based on T-cell recruitment (Fig. 1g, h, Additional file 3: Fig. S2A–B), and China-initiated or involved is concentrated on double immune checkpoint blocking (Fig. 1i, Additional file 3: Fig. S2A–B). Globally, the focus of MOA lies in T-cell recruitment and double immune checkpoint blocking, while investigations into tumor-targeted immunomodulators are rare (Additional file 3: Fig. S2C). In contrast to the focus on T-cell redirection by international pharmaceutical companies (Additional file 3: Fig. S2C), the research trend on the mechanism of double immune checkpoint blocking action has gradually increased in China (Additional file 3: Fig. S2D).

## Diverse targets of bispecific antibodies clinical research

The targets of bsAbs research are drawn into network diagrams (Fig. 1j–l). Obviously, international researches and those in China focus on different targets. International R&D companies mainly focus on CD3-based (*n* = 63) bsAbs (Fig. 1k), while Chinese R&D enterprises pay more attention to PD-1 (*n* = 9)/PD-L1 (*n* = 7) axises compounds (Fig. 1l).

## Diverse cancer types studied in anticancer bispecific antibodies

**Fig. 2:**
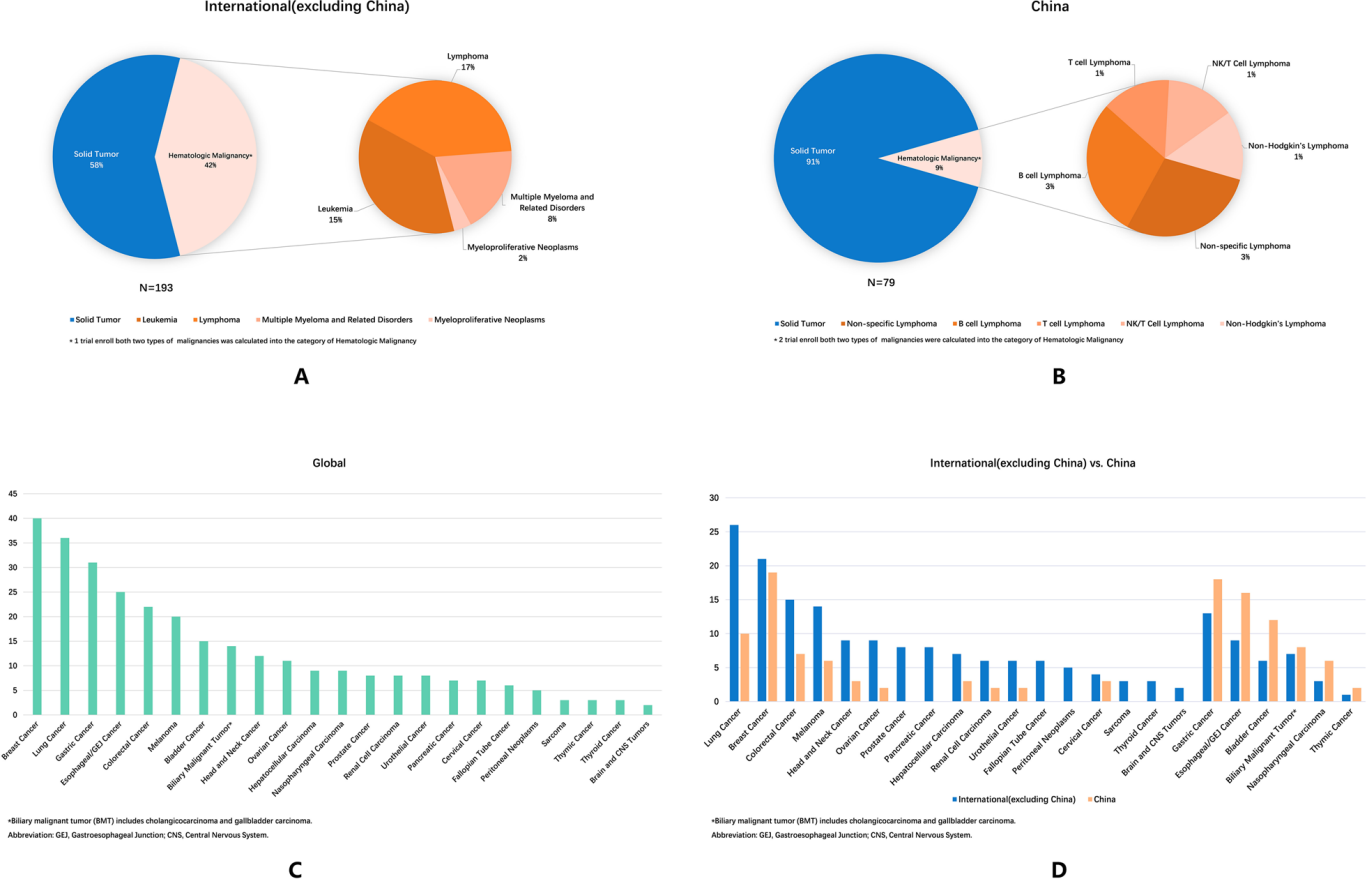
Distribution of studied tumor types. **a** Tumor types of international companies. **b** Tumor types of China-initiated or involved companies. **c** Solid tumor types of overall clinical trials. **d** Solid tumor types of international companies and China-initiated or involved companies, respectively

The investigated tumor-type distribution of bsAbs studies is significantly different between China and other countries (Fig. 2). For international bsAbs clinical studies, solid tumors (58%) and hematological tumors (42%) account for nearly half of each, and subtypes of hematological tumors included lymphoma and leukemia (Fig. 2a). Nearly 91% of Chinese bsAbs clinical studies located at solid tumors, and the research on hematological tumors is obviously underappreciated (only 9% with all subtypes turns lymphomas) (Fig. 2b). For participating centers, international bsAbs clinical studies are dominated by multiple-center design (85%), while single-center design (54%) and multiple-center design (46%) are basically the same in China (Fig. 1d, e). For solid tumors distribution, breast cancer (*n* = 40), lung cancer (*n* = 36), and gastric cancer (*n* = 31) are the top three research tumor types (Fig. 2c). Higher focuses on gastric cancer (*n* = 18), esophageal/gastroesophageal junction (GEJ) cancer (*n* = 16), bladder cancer (*n* = 10), biliary malignant tumor (*n* = 8), nasopharyngeal cancer (*n* = 6), and thymic cancer (*n* = 2) were observed in China (Fig. 2d).

The trend of the anticancer bsAbs investigation differed from the international and the domestic in many aspects with respect to the study time span, quantity, scale, tumor species diversity target coverage, and MOA of bsAbs. To narrow the gap with other countries, China has made great efforts for constructing the evolution of the pharmaceutical ecosystem and innovative drug R&D. By combining academia and industry, more advanced scientific discoveries and technological revolution in China are transforming into anti-tumor drugs innovation. Besides, since 2017 the National Medical Products Administration (NMPA) of China has implemented a series of regulatory reforms to support an environment that encourages novel drugs innovation [8–12], which optimized the drug approval management process and speeded up the approval of imported drugs in China. Under the guidance of national policies, increasing domestic pharmaceutical companies have shifted from focusing on generic drugs to "first-in-class" new anti-tumor candidates’ investigations (including the bsAbs). In spite of the above efforts and achievements, China still has a long way to go in terms of novel drug R&D and the anti-tumor bsAbs investigations. China is supposed to reexamine the current distribution of bsAbs targets, to explore more abundant tumor types, and be open-minded and introduced more novel technologies and products. In that way, more promising anticancer bsAbs will come out and more therapeutic strategies will be investigated to eventually bring new hope to cancer patients.

## Supplementary Information

**Additional file 1.** Supplementary Methods and Table S1–6.

**Additional file 2. Fig. S1**: Geographic distribution of anticancer bsAbs clinical trials. (A) Clinical trials of worldwide companies. (B) Clinical trials of China-initiated or involved R&D pharmaceutical companies.

**Additional file 3. Fig. S2**: The MOA of bsAbs clinical trials. (A-B) The number of MOAs of international companies and China-initiated or involved companies, respectively. (C-D) Surface plot of MOA of international companies and China-initiated or involved companies, respectively.

## Data Availability

The datasets used and/or analyzed during the current study are available from the corresponding author on reasonable request.

## Abbreviations

R&D: Research and Development
BsAb: Bispecific antibody
FDA: The US Food and Drug Administration
CNS: Nervous system tumors
GEJ: Gastroesophageal junction
BMT: Biliary malignant tumor
BpAb: Biparatopic antibody
CTLA-4: Cytotoxic T-lymphocyte-associated protein-4
HER2: Human epidermal growth factor receptor 2
LAG-3: Lymphocyte activation gene-3
MOA: Mechanism of action
PD-1: Programmed cell death protein 1
PD-L1: Programmed death ligand 1
TAA: Tumor-associated antigen
TCR: T-cell receptor
TGF-β: Transforming growth factor β

## References

[1] Husain B, Ellerman D (2018). Expanding the boundaries of biotherapeutics with bispecific antibodies. BioDrugs.

[2] Krishnamurthy A, Jimeno A (2018). Bispecific antibodies for cancer therapy: a review. Pharmacol Ther.

[3] Suurs FV, Lub-de Hooge MN, de Vries EGE, de Groot DJA (2019). A review of bispecific antibodies and antibody constructs in oncology and clinical challenges. Pharmacol Ther.

[4] Zhang JYJ, Zhou P (2020). Development of bispecific antibodies in China: overview and prospects. Antibody Ther.

[5] Zhang MY, Lu JJ, Wang L, Gao ZC, Hu H, Ung CO, Wang YT (2015). Development of monoclonal antibodies in China: overview and prospects. Biomed Res Int.

[6] Zhao S, Lv C, Gong JF, Fang WF, Hu XC, Ba Y, Chen XY, Yang ZM, Lin S, Li Z (2019). Challenges in anticancer drug R&D in China. Lancet Oncol.

[7] Labrijn AF, Janmaat ML, Reichert JM, Parren P (2019). Bispecific antibodies: a mechanistic review of the pipeline. Nat Rev Drug Discov.

[8] China Food and Drug Administration. Policies on reforming clinical trial administrations and encouraging drug and medical device innovations. 2017. http://samrcfdagovcn/WS01/CL0087/172568html. Accessed 8 April 2018 **(in Chinese)**.

[9] China Food and Drug Administration. Notice of the general administration of the people’s republic of China on releasing the work program for the reform of the classification of chemical drugs registration. 2016. http://samrcfdagovcn/WS01/CL0087/146603html. Accessed 8 April 2018 **(in Chinese)**.

[10] China Food and Drug Administration. Opinions of the state council regarding deepening reforms on the examination and approval system, encouraging drug and medical device innovations. 2017. http://www.govcn/xinwen/2017-10/08/content_5230105htm. Accessed 8 April 2018 **(in Chinese)**.

[11] China Food and Drug Administration. Decisions about adjustments of regulations on import drug registration and management. 2017. [http://samrcfdagovcn/WS01/CL1031/178363html. Accessed 3 April 2018 **(in Chinese)**.

[12] China Food and Drug Administration. Policies on accelerating reviews and approvals of new drugs and medical devices to encourage drug and medical device innovations. 2017. http://samrcfdagovcn/WS01/CL0087/172567html. Accessed 4 April 2018 **(in Chinese)**.

