# Supplementary material for: Anticancer bispecific antibody R&D advances: a study focusing on research trend worldwide and in China
Source: J Hematol Oncol. 2021 Aug 16;14:124. doi: 10.1186/s13045-021-01126-x (PMC8369643; doi:10.1186/s13045-021-01126-x)
Supplement: Supplementary file 1 — Additional file 1. Supplementary Methods and Table S1–6. [file 13045_2021_1126_MOESM1_ESM.docx]

**Supplementary Methods**

**Data sources**

We retrieved 272 bsAbs research clinical trials in the world through the United States Food and Drug Administration(FDA) clinical trial website[12] and the Chinese drug clinical trial registration and information disclosure platform[13]. The search cutoff is December 30, 2020.

**Search strategy**

Using terms “bispecific antibody”, we retrieved clinical trials through the aforementioned website, and download all research information, including organizations, clinical trial identity numbers, locations, phases, participating centers, conditions, status, enrollments, targets, spectrums of MOA, start date of bsAbs clinical trials. We divide all clinical trials into two categories: International pharmaceutical enterprises and China-initiated or -involved pharmaceutical enterprises, based on the attributes of pharmaceutical companies.

**Statistical analysis**

Statistical analyses were performed using SPSS 25 (IBM, Armonk, NY, USA). The differences of clinical trials between international pharmaceutical enterprises and China-initiated or -involved pharmaceutical enterprises were analyzed by Pearson X^2^ test, Fisher’s exact test. and *P*-values < 0.05 was considered statistically significant for all analyses. Graph networks were constructed using Gephi-0.9.2.

**Figure Legends**

**Figure S1. Geographic distribution of anticancer bsAbs clinical trials.** (A) clinical trials of worldwide companies. (B)clinical trials of china-initiated or -involve R&D pharmaceutical companies.

**Figure S2. The MOA of bsAbs clinical trials.** (A-B) the number of MOA of international companies and china-initiated or -involve companies respectively. (C-D) Surface plot of MOA of international companies and china-initiated or -involve companies respectively.

**Table S1. Registered bispecific antibody anticancer clinical trials conducted by International R&D pharmaceutical enterprises/companies**

| **Antibody** | **Organization** | **Targets** | **Mechanism of action** | **Centre** | **Locations** | **Clinical Trial ID** | **Phases** | | **Enrollment** | **Status** | **Conditions** | **Start Date** |
| --- | --- | --- | --- | --- | --- | --- | --- | --- | --- | --- | --- | --- |
| Dilpacimab, ABT-165 | AbbVie | VEGF × DLL4 | Dual signaling inhibitions | multi-center | United States | NCT01946074 | Phase I | | 100 | Active, not recruiting | Advanced Solid Tumors | 2013/8/8 |
|  |  |  | Dual signaling inhibitions | multi-center | United States | NCT03368859 | Phase II | | 71 | Completed | Cancer | 2018/2/19 |
|  |  |  |  |  | Belgium |  |  |  |  |  |  |  |
|  |  |  |  |  | Canada |  |  |  |  |  |  |  |
|  |  |  |  |  | Korea |  |  |  |  |  |  |  |
|  |  |  |  |  | Spain |  |  |  |  |  |  |  |
|  |  |  |  |  | **China (Taiwan)** |  |  |  |  |  |  |  |
| MP0250 | Molecular Partners AG | VEGF × HGF | Dual signaling inhibitions | multi-center | Austria, Denmark, Germany, Czechia, Italy, Poland | NCT03136653 | Phase I/II | | 33 | Active, not recruiting | Multiple Myeloma in Relapse | 2017/5/23 |
|  |  |  | Dual signaling inhibitions | multi-center | United States | NCT03418532 | Phase I/II | | 40 | Active, not recruiting | EGFR-mutated NSCLC (Disorder) | 2018/3/22 |
|  |  |  | Dual signaling inhibitions | multi-center | Spain, Switzerland, United Kingdom | NCT02194426 | Phase I/II | | 58 | Completed | Neoplasms | 2020/7/14 |
| ABL-001, NOV-1501, TR-009 | ABL Bio, TRIGR Therapeutics | VEGF × DLL4 | Dual signaling inhibitions | multi-center | United States | NCT03595917 | Phase I | | 34 | Recruiting | B-cell Acute Lymphoblastic Leukemia\|Chronic Myeloid Leukemia (CML) in Lymphoid Blast Crisis\|Philadelphia Chromosome Positive Acute Lymphoblastic Leukemia Ph+ ALL | 2018/7/24 |
|  |  |  | Dual signaling inhibitions | multi-center | United States | NCT03578367 | Phase II | | 80 | Recruiting | CML \|Chronic Myelogenous Leukemia\| Leukemia, Myeloid Chronic \|Hematologic Diseases | 2018/11/22 |
|  |  |  |  |  | United Kingdom |  |  |  |  |  |  |  |
|  |  |  |  |  | Spain |  |  |  |  |  |  |  |
|  |  |  |  |  | Portugal |  |  |  |  |  |  |  |
|  |  |  |  |  | Russia |  |  |  |  |  |  |  |
|  |  |  |  |  | Poland |  |  |  |  |  |  |  |
|  |  |  |  |  | Korea |  |  |  |  |  |  |  |
|  |  |  |  |  | Japan |  |  |  |  |  |  |  |
|  |  |  |  |  | Italy |  |  |  |  |  |  |  |
|  |  |  |  |  | Germany |  |  |  |  |  |  |  |
|  |  |  |  |  | France |  |  |  |  |  |  |  |
|  |  |  |  |  | Denmark |  |  |  |  |  |  |  |
|  |  |  |  |  | Czechia |  |  |  |  |  |  |  |
|  |  |  |  |  | Chile |  |  |  |  |  |  |  |
|  |  |  |  |  | Canada |  |  |  |  |  |  |  |
|  |  |  |  |  | Australia |  |  |  |  |  |  |  |
|  |  |  |  |  | Austria |  |  |  |  |  |  |  |
|  |  |  |  |  | **China (Hong Kong, Taiwan)** |  |  |  |  |  |  |  |
|  |  |  | Dual signaling inhibitions | multi-center | United States, United Kingdom, Turkey, Switzerland, Spain, Serbia, Saudi Arabia, Russian, Romania, Netherlands, Mexico, Lebanon, Korea, Japan, Israel, Italy, Hungary, Germany, France, Czechia, Canada, Bulgaria, Australia, Brazil | NCT03106779 | Phase III | | 234 | Active, not recruiting | Chronic Myelogenous Leukemia | 2017/10/26 |
|  |  |  | Dual signaling inhibitions | single-center | United States | NCT04216563 | Phase II | | 40 | Not yet recruiting | Philadelphia Chromosome Negative, BCR-ABL1 Positive Chronic Myelogenous Leukemia | 2020/4/30 |
|  |  |  | Dual signaling inhibitions | single-center | Korea | NCT03292783 | Phase I | | 39 | Unknown | Advanced Solid Tumors | 2017/9/18 |
|  |  |  | Dual signaling inhibitions | multi-center | United States, Spain, Singapore, Netherlands, Korea, Japan, Italy, Australia, Germany, France | NCT02081378 | Phase I | | 330 | Recruiting | Chronic Myelogenous Leukemia\|Philadelphia Chromosome-positive Acute Lymphoblastic Leukemia | 2014/4/24 |
|  |  |  | Dual signaling inhibitions | multi-center | Germany | NCT03906292 | Phase II | | 120 | Recruiting | Chronic Myeloid Leukemia | 2019/8/19 |
| Vanucizumab, RG-7221 | Roche, Harvard Medical School, National Cancer Centre of Singapore | VEGF × ANGP | Dual signaling inhibitions | multi-center | Belgium, Spain, France | NCT01688206 | Phase I | | 132 | Completed | Neoplasms | 2012/10/31 |
|  |  |  | Dual signaling inhibitions | multi-center | United States, United Kingdom, Spain, France, Belgium, Australia, Austria | NCT02141295 | Phase II | | 197 | Terminated | Colorectal Cancer | 2014/6/30 |
|  |  |  | Dual signaling inhibitions | multi-center | United States, Belgium, Denmark, Canada, Italy, Netherlands, Spain | NCT02665416 | Phase I | 94 | | Completed | Advanced/Metastatic Solid Tumors | 2016/1/25 |
| BI-836880 | Boehringer Ingelheim, Sanofi | VEGF × ANGP | Dual signaling inhibitions | multi-center | Spain, France | NCT02689505 | Phase I | | 24 | Completed | Neoplasms | 2016/4/4 |
|  |  |  | Dual signaling inhibitions | multi-center | France | NCT03468426 | Phase I | | 245 | Recruiting | Non-squamous, Non-Small-Cell Lung Cancer \|Neoplasms | 2018/5/3 |
|  |  |  |  |  | United Kingdom |  |  |  |  |  |  |  |
|  |  |  |  |  | Ukraine |  |  |  |  |  |  |  |
|  |  |  |  |  | Spain |  |  |  |  |  |  |  |
|  |  |  |  |  | Russia |  |  |  |  |  |  |  |
|  |  |  |  |  | Poland |  |  |  |  |  |  |  |
|  |  |  |  |  | Korea |  |  |  |  |  |  |  |
|  |  |  |  |  | Germany |  |  |  |  |  |  |  |
|  |  |  |  |  | United States |  |  |  |  |  |  |  |
|  |  |  |  |  | Australia |  |  |  |  |  |  |  |
|  |  |  |  |  | **China (Hong Kong, Taiwan)** |  |  |  |  |  |  |  |
|  |  |  | Dual signaling inhibitions | multi-center | France, Germany | NCT02674152 | Phase I | | 29 | Active, not recruiting | Neoplasms | 2016/1/5 |
|  |  |  | Dual signaling inhibitions | multi-center | Japan, Germany | NCT03972150 | Phase I | | 21 | Recruiting | Neoplasms | 2019/6/12 |
| Navicixizumab, OMP-305B83 | OncoMed Pharmaceuticals | VEGF × DLL4 | Dual signaling inhibitions | multi-center | United States | NCT03035253 | Phase I | | 16 | Terminated | Metastatic Colorectal Cancer | 2016/12/1 |
|  |  |  | Dual signaling inhibitions | multi-center | United States | NCT03030287 | Phase I | | 44 | Completed | Cancer Ovaries\|Cancer Peritoneal\|Cancer, Fallopian Tube | 2016/12/1 |
|  |  |  | Dual signaling inhibitions | multi-center | United States, Canada, Spain | NCT02298387 | Phase I | | 71 | Completed | Advanced Solid Tumor Malignancies | 2014/12/22 |
| MCLA-128 | Merus | HER2 × HER3 | Dual signaling inhibitions | multi-center | United States, United Kingdom, United Kingdom, Portugal, Netherlands, France, Belgium | NCT03321981 | Phase II | | 120 | Recruiting | Breast Cancer Metastatic | 2018/1/15 |
|  |  |  | Dual signaling inhibitions | multi-center | United States | NCT02912949 | Phase I/II | | 250 | Recruiting | Solid Tumours Harboring NRG1 Fusion \|NSCLC Harboring NRG1 Fusion \|Pancreatic Cancer Harboring NRG1 Fusion\|NRG1 Fusion | 2015/1/1 |
|  |  |  |  |  | Canada |  |  |  |  |  |  |  |
|  |  |  |  |  | France |  |  |  |  |  |  |  |
|  |  |  |  |  | Italy |  |  |  |  |  |  |  |
|  |  |  |  |  | Japan |  |  |  |  |  |  |  |
|  |  |  |  |  | Korea |  |  |  |  |  |  |  |
|  |  |  |  |  | Netherlands |  |  |  |  |  |  |  |
|  |  |  |  |  | Singapore |  |  |  |  |  |  |  |
|  |  |  |  |  | Spain |  |  |  |  |  |  |  |
|  |  |  |  |  | Norway |  |  |  |  |  |  |  |
|  |  |  |  |  | **China (Taiwan)** |  |  |  |  |  |  |  |
| BI-905677 | Boehringer Ingelheim | LRP5 × LRP6 | Biparatopic bsAbs | multi-center | United States, Spain, Netherlands, Japan | NCT03604445 | Phase I | | 60 | Recruiting | Neoplasms | 2018/8/8 |
| MP0274 | Molecular Partners AG | HER2 × HER2 | Biparatopic bsAbs | multi-center | Germany, Switzerland, United Kingdom | NCT03084926 | Phase I | | 46 | Recruiting | Neoplasms | 2017/8/8 |
| DuoBody-PD-L1x4-1BB, GEN-1046 | BioNTech, Genmab | PD-L1 × 4-1BB | Co-localized blockage | multi-center | United States, Israel, Spain | NCT03917381 | Phase I/II | | 192 | Recruiting | Solid Tumors\|Non-small Cell Lung Cancer\|Urothelial Carcinoma\|Endometrial Carcinoma\|Triple Negative Breast Cancer\|Squamous Cell Carcinoma of the Head and Neck\|Cervical Cancer | 2019/5/14 |
| REGN-5678 | Regeneron | PSMA × CD28 | T-cell redirection | multi-center | United States | NCT03972657 | Phase I/II | | 123 | Recruiting | Metastatic Castration-resistant Prostate Cancer | 2019/8/12 |
| FS118 mAb2, FS-118, LAG-3/PD-L1 mAb2 | F-star | PD-L1 × LAG-3 | Dual checkpoint blockade | multi-center | United States | NCT03440437 | Phase I | | 43 | Active, not recruiting | Advanced Cancer\|Metastatic Cancer | 2018/4/16 |
| LY-3434172 | Eli Lilly | PD-1 × PD-L1 | Dual checkpoint blockade | multi-center | United States, France, Korea, Australia, Belgium | NCT03936959 | Phase I | | 40 | Active, not recruiting | Advanced Cancer | 2019/5/24 |
| XmAb-23104 | Xencor | PD-1 × ICOS | Co-localized blockage | multi-center | United States | NCT03752398 | Phase I | | 164 | Recruiting | Melanoma (Excluding Uveal Melanoma)\|Cervical Carcinoma\|Pancreatic Carcinoma\|Breast Carcinoma That is Estrogen Receptor, Progesterone Receptor, and Her2 Negative\|Hepatocellular Carcinoma\|Urothelial Carcinoma\|Squamous Cell Carcinoma of the Head and Neck\|Nasopharyngeal Carcinoma\|Renal Cell Carcinoma\|Colorectal Carcinoma\|Endometrial Carcinoma\|Non-small Cell Lung Carcinoma\|Small Cell Lung Cancer\|Gastric or Gastroesophageal Junction Adenocarcinoma\|Advanced Solid Tumors\|Undifferentiated Pleomorphic Sarcoma | 2019/5/1 |
| ADC-1015, ATOR-1015 | Alligator Bioscience | OX40 × CTLA-4 | Co-localized blockage | multi-center | Denmark, Sweden | NCT03782467 | Phase I | | 53 | Recruiting | Solid Tumor\|Neoplasms | 2019/1/30 |
| MCLA-145 | Merus, Incyte | PD-L1 × 4-1BB | Co-localized blockage | multi-center | United States, Belgium | NCT03922204 | Phase I | | 118 | Recruiting | Advanced Cancer\|Solid Tumor, Adult\|B-cell Lymphoma, Adult | 2019/5/8 |
| MGD-019 | MacroGenics | PD-1 × CTLA-4 | Dual checkpoint blockade | multi-center | United States | NCT03761017 | Phase I | | 167 | Recruiting | Solid Tumor, Adult\|Advanced Cancer | 2018/12/12 |
| PRS-343 | Pieris | HER2 × 4-1BB | Dual signaling inhibitions | multi-center | United States | NCT03330561 | Phase I | | 78 | Recruiting | HER2-positive Breast Cancer\|HER2-positive Gastric Cancer\|HER2-positive Bladder Cancer\|HER2-positive Solid Tumor | 2017/9/28 |
|  |  |  | Dual signaling inhibitions | multi-center | United States | NCT03650348 | Phase I | | 70 | Recruiting | HER2-positive Breast Cancer\|HER2-positive Gastric Cancer\|HER2-positive Bladder Cancer\|HER2-positive Solid Tumor | 2018/8/21 |
| RG-7769, RO-7121661 | Roche | PD-1 × TIM-3 | Dual checkpoint blockade | multi-center | United States, Spain, Denmark, France, Korea | NCT03708328 | Phase I | | 300 | Recruiting | Solid Tumors\|Metastatic Melanoma\|Non-small Cell Lung Cancer (NSCLC)\|Small Cell Lung Cancer (SCLC) | 2018/10/15 |
| XmAb-20717 | Xencor | PD-1 × CTLA-4 | Dual checkpoint blockade | multi-center | United States | NCT03517488 | Phase I | | 154 | Recruiting | Melanoma\|Breast Carcinoma\| Hepatocellular Carcinoma \|Urothelial Carcinoma\|Squamous Cell Carcinoma of the Head and Neck\|Renal Cell Carcinoma\|Colorectal Carcinoma\|Non-small Cell Lung Carcinoma\|Gastric or Gastroesophageal Junction Adenocarcinoma\|Endometrial Carcinoma\| Mesothelioma \|Neuroendocrine Carcinoma\|Cervical Cancer\|Small Cell Lung Carcinoma\|Squamous Cell Carcinoma of the Anus\|Castration-Resistant Prostate Carcinoma\|Nasopharyngeal Carcinoma \|Cholangiocarcinoma\|Basal Cell Carcinoma\|Ovarian Carcinoma \|Fallopian Tube Carcinoma\|Thymoma\|Thymic Carcinoma \|Squamous Cell Carcinoma of the Penis\|Vulvar Carcinoma\|Solid Tumors With Published Evidence of Anti-tumor Activity With Anti-PD1/PDL1 and/or Anti-CTLA4-directed Therapy \|Malignant Adnexal Neoplasms\|Non-squamous Cell Salivary Gland Carcinoma | 2018/7/10 |
| XmAb-22841 | Xencor | LAG-3  × CTLA-4 | Dual checkpoint blockade | multi-center | United States | NCT03849469 | Phase I | | 242 | Recruiting | Melanoma\|Cervical Carcinoma\|Pancreatic Carcinoma\|Triple Negative Breast Cancer\|Hepatocellular Carcinoma\|Urothelial Carcinoma\|Squamous Cell Carcinoma of the Head and Neck\|Nasopharyngeal Carcinoma\|Renal Cell Carcinoma\|Non-small Cell Lung Carcinoma\|Small Cell Lung Carcinoma\|Gastric or Gastroesophageal Junction Adenocarcinoma\|Advanced or Metastatic Solid Tumors\|Prostate Carcinoma\|MSI-H\|Mismatch Repair Deficiency\|Epithelial Ovarian Cancer\|Fallopian Tube Cancer\|Primary Peritoneal Carcinoma\|Intrahepatic Cholangiocarcinoma | 2019/5/29 |
| MP0310 | Molecular Partners AG, Amgen | FAP × CD40 | T-cell redirection | multi-center | France | NCT04049903 | Phase I | | 54 | Recruiting | Advanced Solid Tumor | 2019/9/2 |
| GEN-1042 | BioNTech; Genmab | CD40 × 4-1BB | T-cell redirection | multi-center | United States, Denmark, United Kingdom | NCT04083599 | Phase I/II | | 126 | Recruiting | Malignant Solid Tumor\|Non-Small Cell Lung Cancer\|Colorectal Cancer\|Melanoma | 2019/9/17 |
| AGEN-1423, GS-1423 | Agenus, Gilead | CD73 × TGF-β | T-cell redirection | multi-center | United States | NCT03954704 | Phase I | | 192 | Recruiting | Advanced Solid Tumors | 2019/6/3 |
| Tebentafusp (IMCgp100) | Immunocore | CD3 × HLA-Related | T-cell redirection | single-center | United States | NCT03070392 | Phase II | | 327 | Recruiting | Uveal Melanoma | 2017/10/16 |
|  |  |  | T-cell redirection | single-center | United States | NCT02889861 | Phase II | | 3 | Terminated | Malignant Melanoma | 2017/1/11 |
|  |  |  | T-cell redirection | single-center | United States | NCT02535078 | Phase I/II | | 183 | Recruiting | Malignant Melanoma | 2015/11/1 |
|  |  |  | T-cell redirection | single-center | United States | NCT01209676 | Phase I | | 1 | Completed | Melanoma\|Advanced Disease\|Unresectable | 2010/9/1 |
|  |  |  | T-cell redirection | multi-center | United States, Canada, United Kingdom, Germany, Spain | NCT02570308 | Phase I/II | | 150 | Active, not recruiting | Uveal Melanoma | 2016/2/1 |
|  |  |  | T-cell redirection | multi-center | United States, United Kingdom | NCT01211262 | Phase I | | 84 | Completed | Malignant Melanoma | 2010/9/28 |
| OXS-1550, DT-2219 | GT Biopharma | CD19 × CD22 | T-cell redirection | single-center | United States | NCT02370160 | Phase I/II | | 18 | Completed | Refractory B-Lineage Leukemia\|Relapsed B-Lineage Leukemia\|Refractory B-Lineage Lymphoma\|Relapsed B-Lineage Lymphoma | 2015/12/21 |
|  |  |  | T-cell redirection | multi-center | United States | NCT00889408 | Phase I | | 25 | Completed | Leukemia\|Lymphoma | 2013/12/2 |
| AFM-13 | Affimed | CD16 × CD30 | T-cell redirection | single-center | Germany | NCT02321592 | Phase II | | 23 | Active, not recruiting | Hodgkin Lymphoma | 2015/5/1 |
|  |  |  | T-cell redirection | multi-center | United States, Germany | NCT01221571 | Phase I | | 28 | Completed | Hodgkin Lymphoma | 2010/10/1 |
|  |  |  | T-cell redirection | single-center | United States | NCT03192202 | Phase I/II | | 18 | Recruiting | Lymphoma, T-Cell, Cutaneous | 2017/7/17 |
|  |  |  | T-cell redirection | single-center | United States | NCT04074746 | Phase I | | 30 | Not yet recruiting | Recurrent Anaplastic Large Cell Lymphoma\|Recurrent B-Cell Non-Hodgkin Lymphoma\|Recurrent Classic Hodgkin Lymphoma\|Recurrent Mycosis Fungoides\|Recurrent Peripheral T-Cell Lymphoma\|Refractory Anaplastic Large Cell Lymphoma\|Refractory B-Cell Non-Hodgkin Lymphoma\|Refractory Classic Hodgkin Lymphoma\|Refractory Mycosis Fungoides\|Refractory Peripheral T-Cell Lymphoma\|TNFRSF8 Positive | 2020/4/15 |
|  |  |  | T-cell redirection | multi-center | United States, Turkey, Spain, Russian, Poland, Korea, Italy, Germany, France, Australia | NCT04101331 | Phase II | | 145 | Recruiting | Peripheral T Cell Lymphoma\|Transformed Mycosis Fungoides | 2019/11/13 |
|  |  |  | T-cell redirection | multi-center | United States, Spain | NCT02665650 | Phase I | | 30 | Completed | Hodgkin Lymphoma | 2016/5/1 |
| Odronextamab, REGN-1979 | Regeneron | CD3 × CD20 | T-cell redirection | multi-center | United States, Germany, Spain | NCT02651662 | Phase I | | 172 | Active, not recruiting | Lymphoma | 2016/1/11 |
|  |  |  | T-cell redirection | multi-center | United States, Germany | NCT02290951 | Phase I | | 480 | Recruiting | Non-Hodgkin Lymphoma\|Chronic Lymphocytic Leukemia | 2015/1/9 |
|  |  |  | T-cell redirection | multi-center | United States | NCT03888105 | Phase II | | 497 | Recruiting | B-cell Non-Hodgkin Lymphoma (NHL) | 2019/11/13 |
|  |  |  |  |  | United Kingdom |  |  |  |  |  |  |  |
|  |  |  |  |  | Spain |  |  |  |  |  |  |  |
|  |  |  |  |  | Singapore |  |  |  |  |  |  |  |
|  |  |  |  |  | Poland |  |  |  |  |  |  |  |
|  |  |  |  |  | Korea |  |  |  |  |  |  |  |
|  |  |  |  |  | Italy |  |  |  |  |  |  |  |
|  |  |  |  |  | Germany |  |  |  |  |  |  |  |
|  |  |  |  |  | France |  |  |  |  |  |  |  |
|  |  |  |  |  | Canada |  |  |  |  |  |  |  |
|  |  |  |  |  | Australia |  |  |  |  |  |  |  |
|  |  |  |  |  | **China (Taiwan)** |  |  |  |  |  |  |  |
| IMC-C103C | Genentech; Immunocore | CD3 × HLA-Related | T-cell redirection | multi-center | United States, Spain, United Kingdom | NCT03973333 | Phase I/II | | 144 | Recruiting | Select Advanced Solid Tumors | 2019/5/17 |
| IMCnyeso | GlaxoSmithKline; Immunocore | CD3 × HLA-Related | T-cell redirection | multi-center | United States, Canada，United Kingdom | NCT03515551 | Phase I/II | | 63 | Recruiting | Select Advanced Solid Tumors | 2018/6/15 |
| Mosunetuzumab, RG-7828 | Genentech, Roche, Chugai | CD3 × CD20 | T-cell redirection | multi-center | United States, Canada, | NCT03671018 | Phase I/II | | 262 | Recruiting | B-cell Non-Hodgkin Lymphoma | 2018/9/25 |
|  |  |  | T-cell redirection | multi-center | Australia | NCT04313608 | Phase I | | 20 | Recruiting | B-cell Lymphoma | 2020/5/19 |
|  |  |  | T-cell redirection | multi-center | United States, Austria, Korea, Poland, Spain | NCT03677141 | Phase I/II | | 160 | Recruiting | B-cell Non-Hodgkin Lymphoma | 2019/2/8 |
|  |  |  | T-cell redirection | multi-center | France, Spain, United Kingdom | NCT04246086 | Phase I | | 27 | Not yet recruiting | Follicular Lymphoma | 2020/9/1 |
|  |  |  | T-cell redirection | multi-center | United States, Israel, Poland, Spain | NCT03677154 | Phase I/II | | 92 | Recruiting | Diffuse Large B-cell Lymphoma | 2019/5/23 |
|  |  |  | T-cell redirection | multi-center | United States, United Kingdom, Spain, Korea, Germany, France, Canada, Australia | NCT02500407 | Phase I | | 665 | Recruiting | Lymphocytic Leukemia, Chronic\|Lymphoma, Non Hodgkin | 2015/9/30 |
| OXS-3550, CD161533 TriKE | GT Biopharma, Altor BioScience, U. Minnesota | CD16 × CD33 | T-cell redirection | single-center | United States | NCT03214666 | Phase I/II | | 60 | Recruiting | High-risk Myelodysplastic Syndromes\|Acute Myelogenous Leukemia\|Systemic Mastocytosis\|Mast Cell Leukemia | 2020/1/1 |
| GEN-3013 | Genmab | CD3 × CD20 | T-cell redirection | multi-center | France, Italy, United Kingdom, Singapore, Spain, Poland, Netherlands, Korea, Germany, France, Denmark, Canada, Australia | NCT03625037 | Phase I/II | | 110 | Recruiting | Diffuse Large B-cell Lymphoma\|High-grade B-cell Lymphoma\|Primary Mediastinal Large B-cell Lymphoma\|Follicular Lymphoma\|Mantle Cell Lymphoma\|Small Lymphocytic Lymphoma\|Marginal Zone Lymphoma | 2018/6/26 |
| MCLA-117 | Merus | CD3 × CLEC12 | T-cell redirection | multi-center | United States, Belgium, France, Italy, Netherlands | NCT03038230 | Phase I | | 50 | Recruiting | Acute Myelogenous Leukemia\|Acute Myeloid Leukemia | 2016/4/1 |
| Flotetuzumab, MGD-006 | MacroGenics, Servier | CD3 × CD123 | T-cell redirection | single-center | United States | NCT03739606 | Phase II | | 52 | Not yet recruiting | Acute Biphenotypic Leukemia\|Acute Leukemia\|Chronic Myelogenous Leukemia, BCR-ABL1 Positive\|Hairy Cell Leukemia\|Interleukin-3 Receptor Subunit Alpha Positive\|Recurrent Acute Lymphoblastic Leukemia\|Recurrent Blastic Plasmacytoid Dendritic Cell Neoplasm\|Recurrent Chronic Myelogenous Leukemia, BCR-ABL1 Positive\|Recurrent Hematologic Malignancy\|Recurrent Hodgkin Lymphoma\|Refractory Acute Lymphoblastic Leukemia\|Refractory Blastic Plasmacytoid Dendritic Cell Neoplasm\|Refractory Hematologic Malignancy\|Refractory Hodgkin Lymphoma\|Systemic Mastocytosis | 2020/4/10 |
|  |  |  | T-cell redirection | multi-center | United States | NCT04158739 | Phase I | | 47 | Recruiting | Recurrent Acute Myeloid Leukemia\|Refractory Acute Myeloid Leukemia | 2020/1/6 |
| MGD-007 | MacroGenics | CD3 × GPA33 | T-cell redirection | multi-center | United States | NCT03531632 | Phase I/II | | 52 | Active, not recruiting | Colorectal Cancer Metastatic | 2018/6/4 |
|  |  |  | T-cell redirection | multi-center | United States | NCT02248805 | Phase I | | 95 | Completed | Colorectal Carcinoma | 2014/10/1 |
| REGN-4018 | Regeneron, Sanofi | CD3 × MUC16 | T-cell redirection | multi-center | United States | NCT03564340 | Phase I/II | | 366 | Recruiting | Recurrent Ovarian Cancer\|Recurrent Fallopian Tube Cancer\|Recurrent Primary Peritoneal Cancer | 2018/5/21 |
| Cibisatamab, RO-6958688, RG-7802 | Genentech, Roche, Chugai | CD3 × CEA | Biparatopic bsAbs | multi-center | United States, Netherlands, Spain, Denmark, Italy, France, Canada | NCT02650713 | Phase I | | 228 | Completed | Solid Tumors | 2016/1/7 |
|  |  |  | Biparatopic bsAbs | multi-center | United States, Netherlands, Spain, Denmark, Italy, France, Canada | NCT02324257 | Phase I | | 149 | Completed | Solid Tumors | 2014/12/30 |
|  |  |  | Biparatopic bsAbs | multi-center | United States, Denmark, France, Spain | NCT03866239 | Phase I | | 46 | Active, not recruiting | Colorectal Cancer | 2019/5/7 |
|  |  |  | Biparatopic bsAbs | multi-center | United States | NCT03337698 | Phase I/II | | 305 | Recruiting | Carcinoma, Non-Small-Cell Lung | 2018/1/2 |
|  |  |  |  |  | Australia |  |  |  |  |  |  |  |
|  |  |  |  |  | France |  |  |  |  |  |  |  |
|  |  |  |  |  | Israel |  |  |  |  |  |  |  |
|  |  |  |  |  | Korea |  |  |  |  |  |  |  |
|  |  |  |  |  | Spain |  |  |  |  |  |  |  |
|  |  |  |  |  | United Kingdom |  |  |  |  |  |  |  |
|  |  |  |  |  | **China (Taiwan)** |  |  |  |  |  |  |  |
| AMG-701 | Amgen | CD3 × BCMA | T-cell redirection | multi-center | United States, Australia, Canada, Germany, Japan, Netherlands | NCT03287908 | Phase I | | 270 | Recruiting | Relapsed/Refractory Multiple Myeloma | 2017/11/13 |
| AMG-160 | Amgen | CD3 × PSMA | T-cell redirection | multi-center | United States, Australia, Belgium, France, Austria, Japan, Netherlands, Singapore | NCT03792841 | Phase I | | 120 | Recruiting | Metastatic Castration-resistant Prostate Cancer\|Prostate Cancer | 2019/2/5 |
| AMG-330, MT-114 | Amgen | CD3 × CD33 | T-cell redirection | multi-center | United States, Germany, Netherlands | NCT02520427 | Phase I | | 100 | Recruiting | Relapsed/Refractory AML | 2015/8/31 |
| AMG-424 | Amgen | CD3 × CD38 | T-cell redirection | multi-center | United States, Australia | NCT03445663 | Phase I | | 120 | Recruiting | Relapsed/ Refractory Multiple Myeloma | 2018/7/31 |
| AMG-427 | Amgen | CD3 × FLT3 | T-cell redirection | multi-center | United States, Australia, Canada, Germany | NCT03541369 | Phase I | | 105 | Recruiting | Relapsed/Refractory Acute Myeloid Leukemia (AML) | 2018/9/14 |
| AMG-562 | Amgen | CD3 × CD19 | T-cell redirection | multi-center | United States, Canada, Germany | NCT03571828 | Phase I | | 85 | Recruiting | Diffuse Large B-cell Lymphoma(DLBCL)\|Mantle Cell Lymphoma (MCL)\|Follicular Lymphoma (FL) | 2018/10/29 |
| AMG-596 | Amgen | CD3 × EGFRvIII | T-cell redirection | multi-center | United States, Australia, France, Germany, Netherlands, Spain | NCT03296696 | Phase I | | 200 | Recruiting | Glioblastoma or Malignant Glioma | 2018/4/18 |
| AMG-673 | Amgen | CD3 × CD33 | T-cell redirection | multi-center | United States, Australia, Germany | NCT03224819 | Phase I | | 50 | Recruiting | Acute Myeloid Leukemia | 2017/9/7 |
| AMG-757 | Amgen | CD3 × DLL3 | T-cell redirection | multi-center | United States, Australia, Spain, France, Germany, Japan, Netherlands, United Kingdom | NCT03319940 | Phase I | | 162 | Recruiting | Small Cell Lung Carcinoma | 2017/12/26 |
| AMV-564, TandAb T564 | Affimed, Fred Hutch, Amphivena | CD3 × CD33 | T-cell redirection | multi-center | United States | NCT03144245 | Phase I | | 53 | Active, not recruiting | Acute Myeloid Leukemia | 2017/3/20 |
|  |  |  | T-cell redirection | multi-center | United States | NCT04128423 | Phase I | | 116 | Recruiting | Locally Advanced or Metastatic Solid Tumors | 2019/10/9 |
|  |  |  | T-cell redirection | multi-center | United States | NCT03516591 | Phase I | | 80 | Active, not recruiting | Myelodysplastic Syndrome (MDS) | 2018/6/22 |
| APVO-436 | Aptevo | CD3 × CD123 | T-cell redirection | multi-center | United States | NCT03647800 | Phase I | | 108 | Recruiting | AML\|MDS | 2018/12/13 |
| BI-836909, AMG-420 | Amgen, Boehringer Ingelheim | CD3 × BCMA | T-cell redirection | multi-center | France, Germany | NCT02514239 | Phase I | | 43 | Active, not recruiting | Multiple Myeloma | 2015/7/8 |
|  |  |  | T-cell redirection | multi-center | United States, Australia, Belgium, Japan, Switzerland, | NCT03836053 | Phase I | | 23 | Active, not recruiting | Relapsed and/or Refractory Multiple Myeloma | 2019/3/4 |
| RG-6026, RO-7082859 | Roche | CD3 × CD20 | T-cell redirection | multi-center | Denmark, Belgium, Italy, Spain, United Kingdom | NCT03533283 | Phase I | | 140 | Recruiting | Non-Hodgkins Lymphoma | 2018/5/8 |
|  |  |  | T-cell redirection | multi-center | United States, Australia, Canada, France, Germany, Italy, Spain, United Kingdom | NCT03467373 | Phase I | | 172 | Recruiting | B-Cell Lymphoma\|Non-Hodgkin Lymphoma | 2018/3/13 |
|  |  |  | T-cell redirection | multi-center | Australia | NCT04313608 | Phase I | | 20 | Recruiting | B-cell Lymphoma | 2020/5/19 |
|  |  |  | T-cell redirection | multi-center | France, Spain, United Kingdom | NCT04246086 | Phase I | | 27 | Not yet recruiting | Follicular Lymphoma | 2020/9/1 |
|  |  |  | T-cell redirection | multi-center | Austria, Belgium, Denmark, France, Italy, Spain, United Kingdom | NCT04077723 | Phase I | | 207 | Recruiting | Lymphoma, Non-Hodgkin | 2019/8/13 |
|  |  |  | T-cell redirection | multi-center | United States | NCT03075696 | Phase I | | 620 | Recruiting | Non-Hodgkin's Lymphoma | 2017/2/21 |
|  |  |  |  |  | Australia |  |  |  |  |  |  |  |
|  |  |  |  |  | Belgium |  |  |  |  |  |  |  |
|  |  |  |  |  | Czechia |  |  |  |  |  |  |  |
|  |  |  |  |  | Denmark |  |  |  |  |  |  |  |
|  |  |  |  |  | Canada |  |  |  |  |  |  |  |
|  |  |  |  |  | France |  |  |  |  |  |  |  |
|  |  |  |  |  | Finland |  |  |  |  |  |  |  |
|  |  |  |  |  | New Zealand |  |  |  |  |  |  |  |
|  |  |  |  |  | Italy |  |  |  |  |  |  |  |
|  |  |  |  |  | Poland |  |  |  |  |  |  |  |
|  |  |  |  |  | Spain |  |  |  |  |  |  |  |
|  |  |  |  |  | **China (Taiwan)** |  |  |  |  |  |  |  |
| EM-901, CC-93269 | Celgene | CD3 × BCMA | T-cell redirection | multi-center | United States, Germany, Spain, Sweden | NCT03486067 | Phase I | | 120 | Active, not recruiting | Multiple Myeloma | 2018/4/3 |
| ERY-974 | Chugai | CD3 × GPC3 | T-cell redirection | multi-center | United States, France, Netherlands | NCT02748837 | Phase I | | 29 | Completed | Solid Tumors | 2016/8/1 |
|  |  |  | T-cell redirection | multi-center | United States, Germany | NCT03983395 | Phase I/II | | 158 | Recruiting | Breast Cancer | 2020/2/15 |
| GBR-1342 | Glenmark | CD3 × CD38 | T-cell redirection | multi-center | United States | NCT03309111 | Phase I/II | | 125 | Recruiting | Multiple Myeloma | 2017/10/16 |
| GEM-333 | GEMoaB, Celgene | CD3 × CD33 | T-cell redirection | multi-center | Germany | NCT03516760 | Phase I | | 33 | Recruiting | Acute Myeloid Leukemia\|Relapsed AML\|Refractory AML | 2018/4/11 |
| GEM-3PSCA, GEM3PSCA | GEMoaB, Celgene | CD3 × PSCA | T-cell redirection | multi-center | Germany | NCT03927573 | Phase I | | 24 | Recruiting | Non-small Cell Lung Cancer\|Breast Cancer\|Pancreatic Cancer\|Urogenital Cancer | 2019/4/15 |
| IGM-2323 | IGM Biosciences | CD3 × CD20 | T-cell redirection | multi-center | United States | NCT04082936 | Phase I | | 160 | Recruiting | Non-Hodgkin Lymphoma\|Follicular Lymphoma\|DLBCL\|Mantle Cell Lymphoma\|Marginal Zone Lymphoma | 2019/9/30 |
| JNJ-67571244, JNJ-1244 | Janssen Research & Development | CD3 × CD33 | T-cell redirection | multi-center | United States, Germany, Spain | NCT03915379 | Phase I | | 90 | Recruiting | Leukemia, Myeloid, Acute\|Myelodysplastic Syndromes | 2019/3/28 |
| JNJ-63709178, JNJ-9178 | Janssen Research & Development | CD3 × CD123 | T-cell redirection | multi-center | United States, Spain | NCT02715011 | Phase I | | 120 | Recruiting | Leukemia, Myeloid, Acute | 2016/6/1 |
| JNJ-64007957, JNJ-7957 | Janssen Research & Development | CD3 × BCMA | T-cell redirection | multi-center | United States, Sweden, France, Netherlands, Spain | NCT03145181 | Phase I | | 160 | Recruiting | Hematological Malignancies | 2017/5/16 |
| JNJ-63898081, JNJ-8081 | Janssen Research & Development | CD3 × PSMA | T-cell redirection | multi-center | United States, Canada | NCT03926013 | Phase I | | 90 | Recruiting | Neoplasms | 2019/5/1 |
| Orlotamab, MGD-009 | MacroGenics | CD3 × B7-H3 | T-cell redirection | multi-center | United States | NCT03406949 | Phase I | | 25 | Active, not recruiting | Advanced Solid Tumors | 2018/2/27 |
|  |  |  | T-cell redirection | multi-center | United States, Australia, Canada | NCT02628535 | Phase I | | 67 | Terminated | Mesothelioma\|Bladder Cancer\|Melanoma\|Squamous Cell Carcinoma of the Head and Neck\|Non Small Cell Lung Cancer\|Clear Cell Renal Cell Carcinoma\|Ovarian Cancer\|Thyroid Cancer\|Breast Cancer\|Pancreatic Cancer\|Prostate Cancer\|Colon Cancer\|Soft Tissue Sarcoma | 2015/9/1 |
| Pasotuxizumab, AMG-212, (BAY2010112/MT112) | Amgen, Bayer | CD3 × PSMA | T-cell redirection | multi-center | Austria, Germany | NCT01723475 | Phase I | | 47 | Completed | Prostatic Neoplasms | 2012/11/2 |
| PF-06671008 | Pfizer | CD3 × CDH3 | T-cell redirection | multi-center | United States | NCT02659631 | Phase I | | 28 | Terminated | Neoplasms | 2016/4/28 |
| PF-06863135, PF-3135 | Pfizer | CD3 × BCMA | T-cell redirection | multi-center | United States, Canada | NCT03269136 | Phase I | | 80 | Recruiting | Multiple Myeloma | 2017/11/29 |
| REGN-5458 | Regeneron, Sanofi | CD3 × BCMA | T-cell redirection | multi-center | United States, Belgium | NCT03761108 | Phase I/II | | 74 | Recruiting | Multiple Myeloma | 2019/1/23 |
| RG-6194, BTRC-4017A | Genentech | CD3 × HER2 | T-cell redirection | multi-center | United States, Korea, Australia, Spain | NCT03448042 | Phase I | | 449 | Recruiting | Solid Tumors | 2018/6/6 |
| TNB-383B | TeneoBio, AbbVie | CD3 × BCMA | T-cell redirection | multi-center | United States | NCT03933735 | Phase I | | 72 | Recruiting | Multiple Myeloma | 2019/6/24 |
| XmAb-13676, THG-338 | Xencor | CD3 × CD20 | T-cell redirection | multi-center | United States, France | NCT02924402 | Phase I | | 66 | Recruiting | B-cell Non-Hodgkins Lymphoma\|Chronic Lymphocytic Leukemia\|Small Lymphocytic Lymphoma | 2016/10/1 |
| XmAb-14045, SQZ-622 | Xencor, Novartis | CD3 × CD123 | T-cell redirection | multi-center | United States | NCT02730312 | Phase I | | 145 | Recruiting | Acute Myelogenous Leukemia\|B-cell Acute Lymphoblastic Leukemia\|Blastic Plasmacytoid Dendritic Cell Neoplasm\|Chronic Myeloid Leukemia, Blast Crisis | 2016/8/1 |
| XmAb-18087, XENP-18087 | Xencor | CD3 × SSTR2 | T-cell redirection | multi-center | United States | NCT03411915 | Phase I | | 87 | Recruiting | Neuroendocrine Tumor\|Gastrointestinal Neoplasm | 2018/1/22 |
| HPN-424 | Harpoon | CD3 × PSMA | T-cell redirection | multi-center | United States, United Kingdom | NCT03577028 | Phase I | | 40 | Recruiting | Advanced Prostate Cancer | 2018/7/31 |
| JNJ-64407564 | Janssen | CD3 × GPRC5D | T-cell redirection | multi-center | United States, Belgium, Netherlands, Spain | NCT03399799 | Phase I | | 185 | Recruiting | Hematological Malignancies | 2017/12/16 |
|  |  |  | T-cell redirection | multi-center | United States, Canada, Germany, Netherlands, Spain | NCT04108195 | Phase I | | 100 | Recruiting | Multiple Myeloma | 2020/2/21 |
|  |  |  | T-cell redirection | multi-center | United States, Germany, France, Italy, Spain, United Kingdom, Belgium, Canada, Netherlands | NCT04557098 | Phase I/II | | 228 | Recruiting | Hematological Malignancies | 2020/9/17 |
|  |  |  | T-cell redirection | multi-center | Israel, Spain | NCT04586426 | Phase I | | 48 | Not yet recruiting | Multiple Myeloma | 2020/12/2 |
|  |  |  | T-cell redirection | multi-center | United States, Spain, Poland, Belgium, Germany, Israel, Korea, Netherlands, France | NCT04634552 | Phase I/II | | 158 | Not yet recruiting | Hematological Malignancies | 2020/11/18 |
| RG-6160 (BFCR4350A) | Genentech | CD3 × FcRH5 | T-cell redirection | multi-center | United States, Australia, Canada, Spain | NCT03275103 | Phase I | | 130 | Recruiting | Multiple Myeloma | 2017/9/19 |
| NI-1701, TG-1801 | NovImmune, TG Therapeutics | CD19 × CD47 | Tumor-targeted immunomodulators | multi-center | Australia | NCT03804996 | Phase I | | 16 | Recruiting | B-Cell Lymphoma | 2019/3/5 |
| MCLA-158 | Merus | EGFR × LGR5 | Dual signaling inhibitions | multi-center | Belgium, United States, Spain, France | NCT03526835 | Phase I | | 120 | Recruiting | Advanced/Metastatic Solid Tumors\|Colorectal Cancer | 2018/5/2 |
| ZW-49 | Zymeworks | HER2 × HER2 | Biparatopic bsAbs | multi-center | United States, Canada | NCT03821233 | Phase I | | 150 | Recruiting | HER2-expressing Cancers | 2019/1/24 |
| SAR-440234 | Sanofi | CD3 × CD123 | T-cell redirection | multi-center | United States, France | NCT03594955 | Phase I/II | | 77 | Recruiting | Leukaemia | 2018/10/24 |
| AFM-11 | Affimed | CD3 × CD19 | T-cell redirection | multi-center | Austria, Poland, Czechia, Israel, Russian | NCT02848911 | Phase I | | 17 | Terminated | Leukemia, B-Cell | 2016/10/1 |
|  |  |  | T-cell redirection | multi-center | United States, Czechia, Germany, Poland | NCT02106091 | Phase I | | 16 | Terminated | Relapsed B-Cell Non-Hodgkin Lymphoma\|Refractory B-Cell Non-Hodgkin Lymphoma | 2014/4/1 |
| AFM-24 | Affimed | EGFR × CD16 | T-cell redirection | single-center | United States | NCT04259450 | Phase I/II | | 70 | Recruiting | Advanced Solid Tumor | 2020/4/7 |
| CCW-702 | CIBR*; Scripps | CD3 × PSMA | T-cell redirection | single-center | United States | NCT04077021 | Phase I | | 70 | Recruiting | Castration-Resistant Prostatic Cancer | 2020/6/1 |
| HPN-217 | Harpoon | CD3 × BCMA | T-cell redirection | multi-center | United States | NCT04184050 | Phase I/II | | 70 | Recruiting | Multiple Myeloma in Relapse\|Multiple Myeloma\|Multiple Myeloma of Bone\|Multiple Myeloma With Failed Remission | 2020/3/1 |
| BI-905711 | Boehringer Ingelheim | Cadherin-17  × TRAIL-R2 | T-cell redirection | single-center | Japan | NCT04137289 | Phase I | | 140 | Suspended | Gastrointestinal Neoplasms\|Cholangiocarcinoma\|Pancreatic Neoplasms | 2020/3/11 |
| MT110 | Amgen Research (Munich) GmbH | CD3 × EpCAM | T-cell redirection | multi-center | Germany | NCT00635596 | Phase I | | 65 | Completed | Solid Tumors | 2008/3/1 |
| REGN5093 | Regeneron Pharmaceuticals | c-MET × c-MET | Biparatopic bsAbs | multi-center | United States | NCT04077099 | Phase I/II | | 102 | Recruiting | NSCLC | 2020/1/7 |
| Hu3F8 | Memorial Sloan Kettering Cancer Center | CD3 × GD2 | T-cell redirection | single-center | United States | NCT03860207 | Phase I/II | | 30 | Recruiting | Neuroblastoma\|Osteosarcoma\|Other Solid Tumor Cancers，Sarcoma | 2019/2/22 |
| MDX447 | Dartmouth-Hitchcock Medical Center | EGFR× CD64 | T-cell redirection | single-center | United States | NCT00005813 | phase I | | 13 | Completed | Brain and Central Nervous System Tumors | 1997/3/1 |
| RO7247669 | Hoffmann-La Roche | PD-1 ×LAG-3 | Dual checkpoint blockade | multi-center | Denmark, United Kingdom, Spain, Korea, Portugal, Israel | NCT04140500 | Phase I | | 320 | Recruiting | Solid Tumors\|Metastatic Melanoma\|Non-small Cell Lung Cancer\|Esophageal Squamous Cell Carcinoma | 2019/11/11 |
| CDX-527 | Celldex Therapeutics | PD-L1×CD27 | T-cell redirection | multi-center | United States | NCT04440943 | Phase I | | 96 | Recruiting | Non-small Cell Lung Cancer\|Breast Cancer\|Gastric Cancer\|Renal Cell Carcinoma\|Ovarian Cancer\|Primary Peritoneal Carcinoma\|Fallopian Tube Cancer\|Cholangiocarcinoma\|Bladder Urothelial Carcinoma\|MSI-H Colorectal Cancer\|Esophageal Cancer\|Hepatic Cancer\|Head and Neck Cancer\|Other Solid Tumors | 2020/8/4 |
| rM28 | University Hospital Tuebingen | CD28 ×HMV-MAA | T-cell redirection | single-center | Germany | NCT00204594 | Phase I | | 1 | Completed | Malignant Melanoma | 2005/10/1 |
| removab | AGO Study Group | CD3 × EpCAM | T-cell redirection | single-center | United States | NCT00189345 | Phase II | | 44 | Completed | Ovarian Cancer\|Fallopian Tube Neoplasms\|Peritoneal Neoplasms | 2004/5/1 |
| Code C2624 | Dartmouth-Hitchcock Medical Center | 4G7×H22 | Dual signaling inhibitions | single-center | United States | NCT00014560 | Phase I | | 3 | Terminated | Leukemia\|Lymphoma | 2000/9/28 |
| LY3415244 | Eli Lilly and Company | PD-L1 ×TIM-3 | Dual checkpoint blockade | multi-center | United States, Belgium, Japan | NCT03752177 | Phase I | | 12 | Terminated | Solid Tumor | 2018/11/22 |
| AGEN1223 | Agenus Inc. | Undisclosed | Unclassified | multi-center | United States | NCT04156100 | Phase I | | 40 | Recruiting | Advanced Solid Tumor，hepatobiliary (liver, gall bladder, and bile duct) and pancreatic cancers | 2019/12/10 |
| MGD006 | MacroGenics | CD3 × CD123 | T-cell redirection | multi-center | United States, France, Germany, Italy, Netherlands, United Kingdom | NCT02152956 | Phase I/II | | 330 | Recruiting | AML | 2014/6/9 |
| FS120 | F-star Beta Limited | OX40 ×CD137 | T-cell redirection | single-center | United States | NCT04648202 | Phase I | | 70 | Recruiting | Advanced Cancer\|Metastatic Cancer | 2020/11/18 |
| REGN5459 | Regeneron Pharmaceuticals | CD3 × BCMA | T-cell redirection | multi-center | United States | NCT04083534 | Phase I | | 56 | Recruiting | Relapsed Multiple Myeloma\|Refractory Multiple Myeloma | 2019/9/26 |
| REGN7075 | Regeneron Pharmaceuticals | EGFR×CD28 | T-cell redirection | single-center | United States | NCT04626635 | Phase I/II | | 312 | Not yet recruiting | Advanced Solid Tumors | 2020/11/17 |
| GEN1044 | Genmab | CD3 × 5T4 | T-cell redirection | multi-center | United States, Spain, Denmark | NCT04424641 | Phase I/II | | 378 | Recruiting | Dose Escalation Part: Locally Advanced or Metastatic Solid Tumor(s)\|Expansion Part: Prostate Cancer\|Expansion Part: Esophageal Cancer\|Expansion Part: Triple Negative Breast Cancer (TNBC)\|Expansion Part: Squamous Cell Carcinoma of Head and Neck (SCCHN)\|Expansion Part: Non-small Cell Lung Cancer (NSCLC)\|Expansion Part: Bladder Cancer\|Expansion Part: Uterine Cancer | 2020/7/15 |
| TF2 | Centre René Gauducheau | CEA × HSG | Biparatopic bsAbs | multi-center | France | NCT02587247 | Phase II | | 11 | Completed | Metastatic Colorectal Cancer | 2016/3/1 |
|  |  |  | Biparatopic bsAbs | single-center | United States | NCT00895323 | phase I | | 9 | Unknown | Colorectal Cancer | 2008/11/1 |
|  |  |  | Biparatopic bsAbs | single-center | Netherlands | NCT00860860 | phase I | | 20 | Completed | Colorectal Neoplasms | 2009/7/1 |
|  |  |  | Biparatopic bsAbs | multi-center | France | NCT01221675 | Phase I/II | | 18 | Completed | Small Cell Lung Cancer\|CEA-expressing Non-Small Cell Lung Carcinoma (NSCLC) | 2011/6/1 |
|  |  |  | Biparatopic bsAbs | single-center | United States | NCT01273402 | Phase I | | 30 | Terminated | Metastatic Colorectal Cancer | 2011/2/1 |
|  |  |  | Biparatopic bsAbs | multi-center | France | NCT01730612 | Phase I/II | | 23 | Completed | HER2 Negative Breast Carcinoma Expressing CEA | 2012/12/1 |
|  |  |  | Biparatopic bsAbs | multi-center | France | NCT01730638 | Phase I/II | | 25 | Completed | Medullary Thyroid Carcinoma | 2013/1/1 |
| LY3164530 | Eli Lilly and Company | EGFR × c-MET | Dual signaling inhibitions | multi-center | United States | NCT02221882 | Phase I | | 29 | Completed | Neoplasms\|Neoplasm Metastasis | 2014/8/1 |
| Catumaxomab | Neovii Biotech | CD3 × EpCAM | T-cell redirection | multi-center | Austria, Germany, Spain, United Kingdom | NCT00464893 | Phase II | | 70 | Completed | Gastric Cancer\|Gastric Adenocarcinoma | 2007/4/1 |
|  |  |  | T-cell redirection | single-center | Germany | NCT00352833 | Phase II | | 40 | Completed | Gastric Cancer\|Gastric Adenocarcinoma | 2006/7/1 |
| FBTA05 | Technische Universität München | CD3 × CD20 | T-cell redirection | single-center | Germany | NCT01138579 | Phase I/II | | 9 | Terminated | Leukemia\|Stem Cell Transplantation | 2010/8/1 |
| CC-1 | German Cancer Research Center | CD3 × PSMA | T-cell redirection | single-center | Germany | NCT04496674 | Phase I/II | | 86 | Not yet recruiting | Lung Cancer Squamous Cell | 2020/9/1 |
|  |  |  | T-cell redirection | multi-center | Germany | NCT04104607 | Phase I | | 86 | Recruiting | Castration-Resistant Prostatic Cancer | 2019/11/15 |
| ES414 | Aptevo Therapeutics | CD3 × PSMA | T-cell redirection | multi-center | United States, Australia | NCT02262910 | Phase I | | 35 | Completed | Prostate Cancer | 2015/1/1 |
| BCA101 | Bicara Therapeutics | EGFR × TGF-β | Co-localized blockage | multi-center | United States, Canada | NCT04429542 | Phase I/II | | 292 | Recruiting | TNBC - Triple-Negative Breast Cancer\|Head and Neck Squamous Cell Carcinoma\|Squamous Cell Carcinoma of Anal Canal\|Uveal Melanoma\|Glioblastoma\|Colorectal Cancer\|Chordoma\|Squamous Cell Carcinoma of the Lung\|KRAS G12D\|KRAS G13D\|EGFR Amplification\|Epithelial Ovarian Cancer\|Hepatocellular Carcinoma\|Anaplastic Thyroid Cancer\|Pancreas Cancer | 2020/6/1 |
| MM-111 | Merrimack Pharmaceuticals | HER2 × HER3 | Dual signaling inhibitions | multi-center | United States | NCT01097460 | Phase I | | 16 | Completed | Breast Neoplasms | 2010/4/1 |
|  |  |  | Dual signaling inhibitions | multi-center | United States | NCT00911898 | Phase I | | 20 | Completed | Her2 Amplified Solid Tumors\|Metastatic Breast Cancer | 2009/6/1 |
| MT103 | Amgen | CD3 × CD19 | T-cell redirection | multi-center | Germany | NCT00560794 | Phase II | | 21 | Completed | Acute Lymphoblastic Leukemia | 2008/1/1 |
|  |  |  | T-cell redirection | multi-center | United States, Australia, Italy, United Kingdom, Spain, France | NCT01466179 | Phase II | | 225 | Completed | Acute Lymphoblastic Leukemia | 2011/12/1 |
|  |  |  | T-cell redirection | multi-center | United States, France, Germany, Italy, Netherlands, Australia | NCT01471782 | Phase II | | 93 | Completed | Acute Lymphoblastic Leukemia | 2012/1/1 |
|  |  |  | T-cell redirection | multi-center | Germany | NCT01741792 | Phase II | | 25 | Completed | Diffuse Large B-cell Lymphoma | 2012/7/1 |
|  |  |  | T-cell redirection | multi-center | Australia, Belgium, France, Germany, Italy, Spain, Netherlands, Poland, Romania, Russian, United Kingdom | NCT01207388 | Phase II | | 116 | Completed | B-cell Acute Lymphoblastic Leukemia | 2010/11/1 |
|  |  |  | T-cell redirection | multi-center | Germany | NCT01209286 | Phase II | | 36 | Completed | B-ALL | 2010/10/1 |
| INBRX-105-1, INBRX-105, ES-101 | Inhibrx | PD-L1 × 4-1BB | Co-localized blockage | multi-center | United States | NCT03809624 | Phase I | | 90 | Recruiting | Metastatic Solid Tumors\|Non-small Cell Lung Cancer\|Melanoma\|Head and Neck Squamous Cell Carcinoma\|Gastric Adenocarcinoma\|Renal Cell Carcinoma\|Urothelial Carcinoma\|Esophageal Adenocarcinoma | 2019/1/30 |
| MGD-013 | MacroGenics, ZAI Lab | PD-1 × LAG-3 | Dual checkpoint blockade | multi-center | United States, Australia, Bulgaria, Poland, Spain, Thailand, Ukraine | NCT03219268 | Phase I | | 375 | Recruiting | Advanced Solid Tumors\|Hematologic Neoplasms\|Gastric Cancer\|Ovarian Cancer\|GastroEsophageal Cancer\|HER2-positive Breast Cancer\|HER2-positive Gastric Cancer | 2017/8/18 |
|  |  |  | Dual checkpoint blockade | single-center | **China (Hong Kong)** | NCT04212221 | Phase I/II | | 300 | Recruiting | Advanced Hepatocellular Carcinoma (HCC) | 2020/4/20 |
| EMB-01, FIT-013a | EpimAb Biotherapeutics | EGFR × c-MET | Dual signaling inhibitions | multi-center | United States | NCT03797391 | Phase I/II | | 73 | Recruiting | Neoplasms \|Neoplasm Metastasis \|Non-Small-Cell Lung Cancer | 2018/12/13 |
|  |  |  |  |  | **China (Guangdong)** |  |  |  |  |  |  |  |
| JNJ-61186372, JNJ-6372 | Janssen | EGFR × c-MET | Dual signaling inhibitions | multi-center | United States | NCT02609776 | Phase I | | 460 | Recruiting | Non-Small-Cell Lung Cancer | 2016/5/24 |
|  |  |  |  |  | Australia |  |  |  |  |  |  |  |
|  |  |  |  |  | Canada |  |  |  |  |  |  |  |
|  |  |  |  |  | France |  |  |  |  |  |  |  |
|  |  |  |  |  | Italy |  |  |  |  |  |  |  |
|  |  |  |  |  | Japan |  |  |  |  |  |  |  |
|  |  |  |  |  | Korea |  |  |  |  |  |  |  |
|  |  |  |  |  | Spain |  |  |  |  |  |  |  |
|  |  |  |  |  | United Kingdom |  |  |  |  |  |  |  |
|  |  |  |  |  | **China (Beijing, Jilin, Hunan, Sichuan, Chongqing, Guangdong, Zhejiang, Anhui, Jiangxi, Jiangsu, Jiangsu, Hubei, Fujian, Henan, Taiwan)** |  |  |  |  |  |  |  |
|  |  |  | Dual signaling inhibitions | multi-center | United States | NCT04077463 | Phase I | | 120 | Recruiting | Carcinoma, Non-Small-Cell Lung | 2019/9/4 |
|  |  |  |  |  | Italy |  |  |  |  |  |  |  |
|  |  |  |  |  | France |  |  |  |  |  |  |  |
|  |  |  |  |  | Germany |  |  |  |  |  |  |  |
|  |  |  |  |  | Japan |  |  |  |  |  |  |  |
|  |  |  |  |  | Korea |  |  |  |  |  |  |  |
|  |  |  |  |  | Puerto Rico |  |  |  |  |  |  |  |
|  |  |  |  |  | Spain |  |  |  |  |  |  |  |
|  |  |  |  |  | **China (Beijing, Jilin, Hunan, Sichuan, Chongqing, Guangdong, Jiangxi, Shandong, Shanghai, Liaoning, Tianjin, Hubei, Shaanxi)** |  |  |  |  |  |  |  |
|  |  |  | Dual checkpoint blockade | multi-center | **China (Guangdong, Zhejiang, Hong Kong)** | NCT04178460 | Phase I | | 59 | Recruiting | Gastric Cancer | 2020/2/3 |
|  |  |  | Dual checkpoint blockade | multi-center | United States | NCT04082364 | Phase II/III | | 850 | Recruiting | Gastric Cancer\|Gastroesophageal Junction Cancer\|HER2-positive Gastric Cancer | 2019/9/30 |
|  |  |  |  |  | Italy |  |  |  |  |  |  |  |
|  |  |  |  |  | Germany |  |  |  |  |  |  |  |
|  |  |  |  |  | Korea |  |  |  |  |  |  |  |
|  |  |  |  |  | Poland |  |  |  |  |  |  |  |
|  |  |  |  |  | Singapore |  |  |  |  |  |  |  |
|  |  |  |  |  | **China (Taiwan)** |  |  |  |  |  |  |  |
|  |  |  | Dual checkpoint blockade | multi-center | **China (Beijing, Jiangsu, Shanghai, Hunan, Henan, Guangdong, Anhui, Zhejiang, Liaoning, Jilin, Fujian, Jiangxi, Sichuan, Heilongjiang, Hong Kong)** | CTR20200549 | Phase II | | 321 | Recruiting | Advanced liver cancer (including hepatocellular carcinoma and intrahepatic cholangiocarcinoma) | 2020/4/3 |
| JNJ-75348780 | Janssen Research & Development, LLC | CD3 × CD22 | T-cell redirection | multi-center | Australia | NCT04540796 | Phase I | | 120 | Not yet recruiting | Lymphoma, Non-Hodgkin \|Leukemia, Lymphocytic, Chronic, B-Cell | 2020/11/20 |
|  |  |  |  |  | Israel |  |  |  |  |  |  |  |
|  |  |  |  |  | Korea |  |  |  |  |  |  |  |
|  |  |  |  |  | **China (Taiwan)** |  |  |  |  |  |  |  |
| Amivantamab | Janssen Research & Development, LLC | EGFR x c-MET | Dual signaling inhibitions | multi-center | Argentina | CTR20202472 | Phase III | | 1200 | Not yet recruiting | Non-small Cell Lung Cancer | 2020/12/8 |
|  |  |  |  |  | Australia |  |  |  |  |  |  |  |
|  |  |  |  |  | Belgium |  |  |  |  |  |  |  |
|  |  |  |  |  | Brazil |  |  |  |  |  |  |  |
|  |  |  |  |  | Canada |  |  |  |  |  |  |  |
|  |  |  |  |  | France |  |  |  |  |  |  |  |
|  |  |  |  |  | Germany |  |  |  |  |  |  |  |
|  |  |  |  |  | Hungary |  |  |  |  |  |  |  |
|  |  |  |  |  | India |  |  |  |  |  |  |  |
|  |  |  |  |  | Israel |  |  |  |  |  |  |  |
|  |  |  |  |  | Italy |  |  |  |  |  |  |  |
|  |  |  |  |  | Japan |  |  |  |  |  |  |  |
|  |  |  |  |  | Korea |  |  |  |  |  |  |  |
|  |  |  |  |  | Malaysia |  |  |  |  |  |  |  |
|  |  |  |  |  | Mexico |  |  |  |  |  |  |  |
|  |  |  |  |  | Netherlands |  |  |  |  |  |  |  |
|  |  |  |  |  | Poland |  |  |  |  |  |  |  |
|  |  |  |  |  | Portugal |  |  |  |  |  |  |  |
|  |  |  |  |  | Russia |  |  |  |  |  |  |  |
|  |  |  |  |  | Spain |  |  |  |  |  |  |  |
|  |  |  |  |  | Thailand |  |  |  |  |  |  |  |
|  |  |  |  |  | Turkey |  |  |  |  |  |  |  |
|  |  |  |  |  | Ukraine |  |  |  |  |  |  |  |
|  |  |  |  |  | United Kingdom |  |  |  |  |  |  |  |
|  |  |  |  |  | United States |  |  |  |  |  |  |  |
|  |  |  |  |  | **China (Shanghai, Hunan, Beijing, Henan, Xinjiang, Heilongjiang, Shandong, Liaoning, Hubei, Guangdong, Zhejiang, Jilin, Chongqing, Shaanxi, Sichuan, Jiangsu, Zhejiang, Taiwan)** |  |  |  |  |  |  |  |
|  |  |  | Dual signaling inhibitions | multi-center | France | CTR20202097 | Phase I | | 138 | Recruiting | Advanced Non-small Cell Lung Cancer | 2020/10/22 |
|  |  |  |  |  | Germany |  |  |  |  |  |  |  |
|  |  |  |  |  | Italy |  |  |  |  |  |  |  |
|  |  |  |  |  | Japan |  |  |  |  |  |  |  |
|  |  |  |  |  | Korea |  |  |  |  |  |  |  |
|  |  |  |  |  | United States |  |  |  |  |  |  |  |
|  |  |  |  |  | Spain |  |  |  |  |  |  |  |
|  |  |  |  |  | **China (Hunan, Sichuan, Chongqing, Taiwan, Tianjin, Beijing, Liaoning, Zhejiang, Jiangxi, Guangdong, Shanghai, Jilin, Shaanxi, Shandong)** |  |  |  |  |  |  |  |
|  |  |  | Dual signaling inhibitions | multi-center | United States, Korea, United Kingdom, Canada | NCT04606381 | Phase I | | 80 | Not yet recruiting | Advanced Solid Malignancies | 2020/11/10 |
| ABBV-428 | AbbVie | CD40 × MSLN | T-cell redirection | multi-center | United States | NCT02955251 | Phase I | | 61 | Completed | Advanced Solid Tumors Cancer | 2016/11/18 |
|  |  |  |  |  | Australia |  |  |  |  |  |  |  |
|  |  |  |  |  | France |  |  |  |  |  |  |  |
|  |  |  |  |  | **China (Taiwan)** |  |  |  |  |  |  |  |
| MEDI-5752 | MedImmune | PD-1 × CTLA-4 | Dual checkpoint blockade | multi-center | United States | NCT03530397 | Phase I | | 188 | Recruiting | Selected Advanced Solid Tumors | 2018/4/24 |
|  |  |  |  |  | Australia |  |  |  |  |  |  |  |
|  |  |  |  |  | France |  |  |  |  |  |  |  |
|  |  |  |  |  | Italy |  |  |  |  |  |  |  |
|  |  |  |  |  | Korea |  |  |  |  |  |  |  |
|  |  |  |  |  | Netherlands |  |  |  |  |  |  |  |
|  |  |  |  |  | Portugal |  |  |  |  |  |  |  |
|  |  |  |  |  | Spain |  |  |  |  |  |  |  |
|  |  |  |  |  | **China (Taiwan)** |  |  |  |  |  |  |  |

Abbreviation: BsAb, bispecific antibody

**Table S2. Registered bispecific antibody anticancer clinical trials conducted by China-initiated or -involve R&D pharmaceutical enterprises/companies**

| **Antibody** | **Organization** | **Targets** | **Mechanism of action** | **Centre** | **Locations** | **Clinical studies** | **Phases** | **Enrollment** | **Status** | **Conditions** | **Start Date** |
| --- | --- | --- | --- | --- | --- | --- | --- | --- | --- | --- | --- |
| KN-026 | Jiangsu Alphamab Biopharmaceuticals | HER2 × HER2 | Biparatopic bsAbs | single-center | **China(Heilongjiang)** | NCT04165993 | Phase II | 70 | Recruiting | Metastatic Breast Cancer | 2019/12/31 |
|  |  |  | Biparatopic bsAbs | single-center | **China (Beijing)** | NCT04040699 | Phase I | 24 | Recruiting | HER2 Positive Solid Tumor | 2019/9/26 |
|  |  |  | Biparatopic bsAbs | single-center | **China (Shanghai)** | NCT03619681 | Phase I | 20 | Recruiting | Breast Cancer \|Gastric Cancer | 2018/9/17 |
|  |  |  | Biparatopic bsAbs | single-center | **China (Beijing)** | NCT03925974 | Phase II | 40 | Recruiting | Gastric/Gastroesophageal Junction Cancer | 2019/6/17 |
|  |  |  | Biparatopic bsAbs | multi-center | **China (Heilongjiang, Hunan, Liaoning, Zhejiang, Shandong, Guangxi, Shandong, Guangdong, Jiangsu)** | CTR20192289 | Phase II | 68 | Recruiting | Metastatic Breast Cancer | 2019/11/25 |
|  |  |  | Biparatopic bsAbs | single-center | United States | NCT03847168 | Phase I | 20 | Recruiting | Breast Cancer\|Gastric/Gastroesophageal Junction Cancer | 2019/6/18 |
|  |  |  | Biparatopic bsAbs | multi-center | **China (Beijing, Heilongjiang, Henan, Zhejiang, Jiangxi, Jiangsu, Hubei, Fujian, Fujian, Jiangsu, Henan, Shanxi, Shandong)** | CTR20190853 | Phase II | 40 | Recruiting | HER2 positive advanced Gastric/Gastroesophageal Junction Cancer | 2019/5/13 |
|  |  |  | Biparatopic bsAbs | single-center | **China (Shanghai)** | CTR20180690 | Phase I | 24 | Recruiting | HER2 positive advanced Breast Cancer and Gastric Cancer | 2018/8/13 |
| KN-046 | Jiangsu Alphamab Biopharmaceuticals Co., Ltd | PD-1× PD-L1 | Dual checkpoint blockade | single-center | **China (Shanghai)** | NCT03838848 | Phase II | 149 | Recruiting | Stage IV Non-small Cell Lung Cancer | 2019/5/5 |
|  |  |  |  |  |  |  |  |  |  |  |  |
|  |  |  | Dual checkpoint blockade | single-center | **China (Jiangsu)** | NCT03927495 | Phase II | 100 | Recruiting | Esophageal Squamous Cell Carcinoma | 2019/5/22 |
|  |  |  | Dual checkpoint blockade | single-center | **China (Beijing)** | NCT03925870 | Phase II | 30 | Recruiting | Esophageal Squamous Cell Carcinoma | 2019/6/21 |
|  |  |  | Dual checkpoint blockade | single-center | **China (Guangdong)** | NCT03733951 | Phase I | 285 | Recruiting | Advanced Solid Tumors \|Lymphoma | 2018/12/18 |
|  |  |  | Dual checkpoint blockade | single-center | **China (Guangdong)** | NCT04054531 | Phase II | 50 | Recruiting | Non-small Cell Lung Cancer | 2019/9/4 |
|  |  |  | Dual checkpoint blockade | single-center | **China (Beijing)** | NCT03872791 | Phase I/II | 90 | Recruiting | Triple-negative Breast Cancer | 2019/5/30 |
|  |  |  | Dual checkpoint blockade | single-center | **China (Beijing)** | NCT04542837 | Phase II | 30 | Not yet recruiting | HCC | 2020/9/11 |
|  |  |  | Dual checkpoint blockade | multi-center | **China (Beijing, Henan, Zhejiang)** | CTR20190427 | Phase II | 30 | Recruiting | Advanced unresectable or metastatic squamous esophageal carcinoma | 2019/5/13 |
|  |  |  | Dual checkpoint blockade | multi-center | **China (Shanghai, Henan, Anhui, Hubei, Shandong, Jiangsu, Henan, Anhui, Guangdong, Heilongjiang, Hubei, Hunan, Sichuan, Shandong, Guangxi, Fujian, Guangdong, Tianjin, Shandong, Shaanxi, Hunan, Zhejiang, Jiangsu, Shandong, Guangxi, Hunan, Liaoning, Henan, Zhejiang, Beijing, Henan, Sichuan, Hubei, Yunnan, Zhejiang, Chongqing, Fujian, Hebei)** | CTR20201294 | Phase III | 516 | Not yet recruiting | Advanced squamous non-small cell lung cancer | 2020/7/27 |
|  |  |  | Dual checkpoint blockade | multi-center | **China (Beijing, Heilongjiang, Hunan, Liaoning, Jilin, Guangdong, Shandong, Shandong)** | CTR20190197 | Phase I/II | 110 | Recruiting | Breast Cancer | 2019/4/16 |
|  |  |  | Dual checkpoint blockade | multi-center | **China (Beijing, Guangdong, Henan, Heilongjiang, Jilin)** | CTR20191219 | Phase II | 50 | Recruiting | Non-small Cell Lung Cancer | 2019/6/25 |
|  |  |  | Dual checkpoint blockade | multi-center | **China (Zhejiang, Henan, Beijing, Fujian, Jiangsu)** | CTR20190195 | Phase II | 149 | Recruiting | Advanced Non-small Cell Lung Cancer | 2019/1/31 |
|  |  |  | Dual checkpoint blockade | multi-center | **China (Beijing, Shanghai, Guangdong)** | CTR20181996 | Phase I | 285 | Recruiting | Advanced Solid Tumor or Lymphoma | 2018/11/2 |
|  |  |  | Dual checkpoint blockade | multi-center | **China (Beijing, Guangdong, Zhejiang, Henan, Fujian, Jiangsu, Shandong, Anhui, Tianjin, Hebei, Shandong, Sichuan, Hunan, Jiangsu, Fujian, Jiangxi, Inner Mongolia, Gansu, Shaanxi)** | CTR20201699 | Phase II | 85 | Not yet recruiting | HER2 Positive Solid Tumor | 2020/8/21 |
|  |  |  | Dual checkpoint blockade | single-center | Australia | NCT03529526 | Phase I | 21 | Unknown | Advanced Solid Tumors | 2018/5/21 |
|  |  |  | Dual checkpoint blockade | single-center | **China (Shanghai)** | NCT04469725 | Phase II | 66 | Not yet recruiting | Thymic Carcinoma | 2020/8/31 |
|  |  |  | Dual checkpoint blockade | multi-center | **China (Shanghai, Beijing, Zhejiang, Yunnan, Hunan, Fujian, Shandong, Henan, Sichuan, Heilongjiang)** | CTR20201006 | Phase I | 116 | Not yet recruiting | Thymic Carcinoma | 2020/6/24 |
|  |  |  | Dual checkpoint blockade | single-center | China（Beijing） | NCT04040699 | Phase I | 24 | Recruiting | HER2 Positive Solid Tumor | 2019/9/26 |
| AK-104 | Akeso Biopharma | PD-1 × CTLA-4 | Dual checkpoint blockade | single-center | **China (Guangdong)** | NCT04172454 | Phase I/II | 120 | Not yet recruiting | Advaced Solid Tumors \|Melanoma | 2019/12/1 |
|  |  |  | Dual checkpoint blockade | single-center | **China (Guangdong)** | NCT04220307 | Phase II | 140 | Not yet recruiting | Nasopharyngeal Carcinoma | 2020/2/10 |
|  |  |  | Dual checkpoint blockade | single-center | **China (Beijing)** | NCT03852251 | Phase I/II | 112 | Recruiting | Gastric Adenocarcinoma \|Advanced Solid Tumors \|Gastroesophageal Junction Adenocarcinoma | 2019/1/18 |
|  |  |  | Dual checkpoint blockade | multi-center | **China (Beijing, Heilongjiang, Shandong, Zhejiang, Jiangsu, Shandong, Henan)** | CTR20200779 | Phase II | 30 | Active, not recruiting | HCC | 2020/5/11 |
|  |  |  | Dual checkpoint blockade | single-center | **China (Jilin)** | CTR20202184 | Phase II | 70 | Active, not recruiting | Locally advanced unresectable or metastatic highly unstable satellite (MSI-H) or mismatch repair defective (dMMR) solid tumor | 2020/2/25 |
|  |  |  | Dual checkpoint blockade | single-center | **China (Guangdong)** | NCT04547101 | Phase II | 70 | Recruiting | MSI-H/dMMR Solid Tumor | 2020/4/24 |
|  |  |  | Dual checkpoint blockade | single-center | **China (Beijing)** | NCT04444167 | Phase I/II | 30 | Recruiting | Hepatocellular Carcinoma | 2020/6/30 |
|  |  |  | Dual checkpoint blockade | single-center | **China (Beijing)** | NCT04444141 | Phase I/II | 80 | Recruiting | Peripheral T-cell Lymphoma | 2020/5/15 |
|  |  |  | Dual checkpoint blockade | single-center | **China (Beijing)** | NCT04556253 | phase II | 29 | Not yet recruiting | MSI-H/dMMR Gastric Carcinoma and Colorectal Cancer | 2020/10/1 |
|  |  |  | Dual checkpoint blockade | multi-center | Australia | NCT03261011 | Phase I | 153 | Recruiting | Advanced Cancer | 2017/10/3 |
| INBRX-105-1, INBRX-105, ES-101 | Elpiscience BioPharma | PD-L1 × 4-1BB | Co-localized blockage | single-center | **China (Shanghai)** | NCT04009460 | Phase I | 180 | Recruiting | Solid Tumors \|Neoplasms \|Malignant Tumor | 2019/6/28 |
| HX-009 | HanX Biopharmaceuticals | PD-1 × CD47 | T-cell redirection | multi-center | **China (Beijing, Henan)** | CTR20192299 | Phase I | 30 | Active, not recruiting | Advanced Solid Tumor | 2019/11/12 |
|  |  |  | T-cell redirection | single-center | Australia | NCT04097769 | Phase I | 37 | Recruiting | Advanced Solid Tumor | 2019/6/12 |
| Bintrafusp alfa(M7824) | HanX Biopharmaceuticals | PD-L1 × TGF-β | Co-localized blockage | multi-center | United States | NCT04246489 | Phase II | 135 | Recruiting | Uterine Cervical Neoplasms | 2020/3/30 |
|  |  |  |  |  | Argentina |  |  |  |  |  |  |
|  |  |  |  |  | Australia |  |  |  |  |  |  |
|  |  |  |  |  | Belgium |  |  |  |  |  |  |
|  |  |  |  |  | Brazil |  |  |  |  |  |  |
|  |  |  |  |  | France |  |  |  |  |  |  |
|  |  |  |  |  | Hungary |  |  |  |  |  |  |
|  |  |  |  |  | Japan |  |  |  |  |  |  |
|  |  |  |  |  | Korea |  |  |  |  |  |  |
|  |  |  |  |  | Russia |  |  |  |  |  |  |
|  |  |  |  |  | Spain |  |  |  |  |  |  |
|  |  |  |  |  | **China (Shanghai, Chongqing, Guangdong, Hubei)** |  |  |  |  |  |  |
|  |  |  | Co-localized blockage | multi-center | United States | NCT04066491 | Phase II/III | 512 | Recruiting | Biliary Tract Cancer \|Cholangiocarcinoma \|Gallbladder Cancer | 2019/9/20 |
|  |  |  |  |  | Argentina |  |  |  |  |  |  |
|  |  |  |  |  | Australia |  |  |  |  |  |  |
|  |  |  |  |  | Brazil |  |  |  |  |  |  |
|  |  |  |  |  | Chile |  |  |  |  |  |  |
|  |  |  |  |  | France |  |  |  |  |  |  |
|  |  |  |  |  | Japan |  |  |  |  |  |  |
|  |  |  |  |  | Korea |  |  |  |  |  |  |
|  |  |  |  |  | Poland |  |  |  |  |  |  |
|  |  |  |  |  | Spain |  |  |  |  |  |  |
|  |  |  |  |  | China (Taiwan) |  |  |  |  |  |  |
|  |  |  | Co-localized blockage | single-center | United States | NCT04220775 | Phase I/II | 21 | Recruiting | Recurrent Head and Neck Squamous Cell Carcinoma\|Second Primary Squamous Cell Carcinoma of the Head and Neck | 2020/3/18 |
|  |  |  | Co-localized blockage | multi-center | United States, Belgium, France | NCT03840915 | Phase I/II | 64 | Recruiting | Carcinoma, Non-Small-Cell Lung | 2019/3/14 |
|  |  |  | Co-localized blockage | multi-center | United States | NCT04396535 | Phase II | 80 | Not yet recruiting | Advanced Lung Non-Small Cell Carcinoma\|Stage III Lung Cancer AJCC v8\|Stage IIIA Lung Cancer AJCC v8\|Stage IIIB Lung Cancer AJCC v8\|Stage IIIC Lung Cancer AJCC v8\|Stage IV Lung Cancer AJCC v8\|Stage IVA Lung Cancer AJCC v8\|Stage IVB Lung Cancer AJCC v8 | 2020/6/1 |
|  |  |  | Co-localized blockage | single-center | **China (Hong Kong)** | NCT04396886 | Phase II | 37 | Recruiting | Nasopharyngeal Carcinoma \|Recurrent Carcinoma \|Metastatic Cancer \|Non-keratinizing Carcinoma | 2020/2/27 |
|  |  |  | Co-localized blockage | multi-center | United States | NCT03631706 | Phase III | 584 | Recruiting | Non-small Cell Lung Cancer | 2018/10/19 |
|  |  |  |  |  | Argentina |  |  |  |  |  |  |
|  |  |  |  |  | Belgium |  |  |  |  |  |  |
|  |  |  |  |  | Brazil |  |  |  |  |  |  |
|  |  |  |  |  | Canada |  |  |  |  |  |  |
|  |  |  |  |  | France |  |  |  |  |  |  |
|  |  |  |  |  | Germany |  |  |  |  |  |  |
|  |  |  |  |  | Greece |  |  |  |  |  |  |
|  |  |  |  |  | Italy |  |  |  |  |  |  |
|  |  |  |  |  | Japan |  |  |  |  |  |  |
|  |  |  |  |  | Korea |  |  |  |  |  |  |
|  |  |  |  |  | Netherlands |  |  |  |  |  |  |
|  |  |  |  |  | Spain |  |  |  |  |  |  |
|  |  |  |  |  | Turkey |  |  |  |  |  |  |
|  |  |  |  |  | Ukraine |  |  |  |  |  |  |
|  |  |  |  |  | **China (Hong Kong, Taiwan)** |  |  |  |  |  |  |
|  |  |  | Co-localized blockage | multi-center | United States | NCT02517398 | Phase I | 600 | Active, not recruiting | Solid Tumors | 2015/8/31 |
|  |  |  |  |  | Australia |  |  |  |  |  |  |
|  |  |  |  |  | Belgium |  |  |  |  |  |  |
|  |  |  |  |  | Canada |  |  |  |  |  |  |
|  |  |  |  |  | France |  |  |  |  |  |  |
|  |  |  |  |  | Germany |  |  |  |  |  |  |
|  |  |  |  |  | Italy |  |  |  |  |  |  |
|  |  |  |  |  | Japan |  |  |  |  |  |  |
|  |  |  |  |  | Korea |  |  |  |  |  |  |
|  |  |  |  |  | Spain |  |  |  |  |  |  |
|  |  |  |  |  | United Kingdom |  |  |  |  |  |  |
|  |  |  |  |  | **China (Taiwan)** |  |  |  |  |  |  |
|  |  |  | Co-localized blockage | multi-center | United States | NCT03840902 | Phase II | 350 | Recruiting | Non-small Cell Lung Cancer | 2019/4/16 |
|  |  |  |  |  | Argentina |  |  |  |  |  |  |
|  |  |  |  |  | Australia |  |  |  |  |  |  |
|  |  |  |  |  | Brazil |  |  |  |  |  |  |
|  |  |  |  |  | Belgium |  |  |  |  |  |  |
|  |  |  |  |  | Canada |  |  |  |  |  |  |
|  |  |  |  |  | France |  |  |  |  |  |  |
|  |  |  |  |  | Czechia |  |  |  |  |  |  |
|  |  |  |  |  | Japan |  |  |  |  |  |  |
|  |  |  |  |  | Korea |  |  |  |  |  |  |
|  |  |  |  |  | Netherlands |  |  |  |  |  |  |
|  |  |  |  |  | Spain |  |  |  |  |  |  |
|  |  |  |  |  | **China (Taiwan)** |  |  |  |  |  |  |
|  |  |  | Co-localized blockage | multi-center | United States | NCT03833661 | Phase II | 141 | Recruiting | Biliary Tract Cancer \|Cholangiocarcinoma \|Gallbladder Cancer | 2019/3/26 |
|  |  |  |  |  | France |  |  |  |  |  |  |
|  |  |  |  |  | Italy |  |  |  |  |  |  |
|  |  |  |  |  | Korea |  |  |  |  |  |  |
|  |  |  |  |  | Japan |  |  |  |  |  |  |
|  |  |  |  |  | Spain |  |  |  |  |  |  |
|  |  |  |  |  | United Kingdom |  |  |  |  |  |  |
|  |  |  |  |  | **China (Beijing, Heilongjiang, Taiwan)** |  |  |  |  |  |  |
| SHR-1701 | Jiangsu Hengrui | PD-L1 × TGF-β | Co-localized blockage | single-center | **China (Beijing)** | NCT03710265 | Phase I | 112 | Recruiting | Solid Tumor | 2018/11/20 |
|  |  |  | Co-localized blockage | multi-center | **China (Anhui, Guangdong, Henan, Hunan, Jiangsu, Jiangxi, Liaoning, Shandong, Zhejiang, Beijing, Chongqing)** | NCT03774979 | Phase I | 238 | Recruiting | Solid Tumor | 2019/1/24 |
|  |  |  | Co-localized blockage | single-center | **China (Guangdong)** | NCT04282070 | Phase I | 40 | Recruiting | Nasopharyngeal Carcinoma | 2020/3/27 |
|  |  |  | Co-localized blockage | multi-center | Australia | NCT04324814 | Phase I | 48 | Recruiting | Advanced Solid Tumor | 2020/3/24 |
|  |  |  |  |  | **China (Guangdong)** |  |  |  |  |  |  |
| EMB-02 | Shanghai EpimAb Biotherapeutics Co., Ltd. | PD-1×LAG-3 | Dual checkpoint blockade | single-center | **China (Shanghai)** | NCT04618393 | Phase I/II | 43 | Not yet recruiting | Advanced Solid Tumors | 2020/12/31 |
| MBS-301 | Beijing Mabworks Biotech | HER2 × HER2 | Biparatopic bsAbs | single-center | **China (Shanghai)** | NCT03842085 | Phase I | 34 | Recruiting | HER2-positive Recurrent or Metastatic Malignant Solid Tumor | 2019/4/11 |
| IBI318 | Innovent Biologics, Lilly | PD-1 ×PD-L1 | Dual checkpoint blockade | single-center | **China (Guangdong)** | NCT04602065 | Phase I/II | 129 | Not yet recruiting | Extranodal NK/T Cell Lymphoma, Nasal Type | 2020/10/15 |
|  |  |  | Dual checkpoint blockade | single-center | **China (Guangdong)** | NCT03875157 | Phase I | 327 | Recruiting |  | 2019/4/19 |
| IBI-322 | Innovent | PD-L1 × CD47 | T-cell redirection | single-center | **China (Beijing)** | NCT04328831 | Phase I | 218 | Not yet recruiting | Advanced Malignancies | 2020/4/1 |
| IBI-315 | Innovent Biologics (Suzhou) Co. Ltd. | PD-1 × HER2 | Tumor-targeted immunomodulators | single-center | **China (Beijing)** | NCT04162327 | Phase I | 191 | Recruiting | Advanced Solid Tumor | 2019/11/26 |
| A-319 | Generon (Shanghai) | CD3 × CD19 | T-cell redirection | single-center | **China (Jiangsu)** | NCT04056975 | Phase I | 54 | Not yet recruiting | Relapsed or Refractory B-cell Lymphoma | 2019/9/15 |
|  |  |  | T-cell redirection | single-center | **China (Beijing)** | CTR20190205 | Phase I | 48 | Recruiting | Relapsed or Refractory B-cell Lymphoma | 2019/4/8 |
| M701 | Wuhan YZY Biopharma Co., Ltd. | CD3 × EpCAM | T-cell redirection | multi-center | **China (Beijing, Hubei)** | NCT04501744 | Phase I | 42 | Recruiting | Malignant Ascites \|Cancer | 2018/10/30 |
|  |  |  | T-cell redirection | multi-center | **China (Beijing, Hubei, Henan)** | CTR20181212 | Phase I | 30 | Recruiting | Malignant ascites | 2018/8/14 |
| M802 | YZY Biopharma | CD3×HER2 | T-cell redirection | single-center | **China (Shanghai)** | CTR20171194 | Phase I | 32 | Recruiting | HER2-positive Advanced Solid Tumor | 2018/7/26 |
|  |  |  | T-cell redirection | single-center | **China (Shanghai)** | NCT04501770 | Phase I | 32 | Recruiting | HER2-Positive Solid Tumors | 2018/9/17 |
| IMM0306 | ImmuneOnco Biopharma | CD20 ×CD47 | Tumor-targeted immunomodulators | multi-center | **China (Beijing, Henan)** | CTR20192612 | Phase I | 42 | Recruiting | Relapsed or Refractory CD20-positive Non-Hodgkin's lymphoma | 2020/3/23 |
| AK112 | Akeso Biopharma | PD-1 × VEGF | Tumor-targeted immunomodulators | single-center | **China (Guangdong)** | NCT04597541 | Phase I/II | 264 | Not yet recruiting | Solid Tumor, Adult | 2020/10/1 |
|  |  |  | Tumor-targeted immunomodulators | multi-center | Australia | NCT04047290 | Phase I | 132 | Not yet recruiting | Neoplasms Malignant | 2019/8/15 |
| SI-B003 | Sichuan Baili Pharmaceutical Co., Ltd. | PD-1 ×CTLA-4 | Dual checkpoint blockade | multi-center | **China (Beijing, Chongqing, Henan, Shanghai)** | NCT04606472 | Phase I | 159 | Recruiting | Non-small cell lung cancer (NSCLC), Renal cell carcinoma (RCC), Urothelial Cancer (UC), Metastatic colorectal cancer (mCRC), Triple negative breast cancer (TNBC); Melanoma | 2020/10/1 |
|  | Sichuan Baili Pharmaceutical Co., Ltd. | PD-1 ×CTLA-4 | Dual checkpoint blockade | multi-center | **China (Beijing, Henan)** | CTR20201358 | Phase I | 159 | Not yet recruiting | Recurrent or metastatic solid tumors (including colorectal cancer, melanoma, non-small cell lung cancer, urothelial carcinoma, triple negative breast cancer, cervical cancer, gastric cancer and advanced renal cell carcinoma) | 2020/8/5 |
| SI-B001 | Sichuan Baili Pharmaceutical Co., Ltd. | EGFR × HER3 | Dual signaling inhibitions | single-center | **China (Guangdong)** | NCT04603287 | Phase I | 96 | Recruiting | Locally Advanced or Metastatic Epithelial Tumor | 2020/5/11 |
|  | Sichuan Baili Pharmaceutical Co., Ltd. | EGFR × HER3 | Dual signaling inhibitions | single-center | **China (Guangdong)** | CTR20200502 | Phase I | 96 | Recruiting | Local advanced or metastatic epithelial tumors | 2020/3/26 |
| ZW-25 | Zymeworks, BeiGene | HER2 × HER2 | Biparatopic bsAbs | multi-center | United States, Spain, Korea | NCT04466891 | Phase II | 100 | Recruiting | HER2-amplified Biliary Tract Cancers | 2020/10/1 |
|  |  |  | Biparatopic bsAbs | multi-center | United States | NCT04224272 | Phase II | 76 | Recruiting | HER2+/HR+ Breast Cancer | 2020/4/1 |
|  |  |  | Biparatopic bsAbs | multi-center | United States, Canada, Korea | NCT02892123 | Phase I | 234 | Recruiting | HER2-expressing Cancers | 2016/9/1 |
|  |  |  | Biparatopic bsAbs | multi-center | United States, Canada, Korea | NCT03929666 | Phase II | 115 | Recruiting | HER2-expressing Gastroesophageal Adenocarcinoma | 2019/4/10 |
|  |  |  | Biparatopic bsAbs | multi-center | Korea | NCT04276493 | Phase I/II | 50 | Recruiting | Breast Cancer \|Gastric Cancer \|Gastroesophageal Junction Cancer | 2020/3/26 |
|  |  |  |  |  | **China (Taiwan)** |  |  |  |  |  |  |
|  |  |  | Biparatopic bsAbs | multi-center | United States, Australia, Netherlands | NCT04380805 | Phase II | 40 | Not yet recruiting | Recurrent Cervical Cancer\|Metastatic Cervical Cancer | 2020/6/1 |
| GBR-1302 | Glenmark, Harbour BioMed | CD3 × HER2 | T-cell redirection | multi-center | United States, Germany | NCT02829372 | Phase I | 36 | Terminated | HER2 Expressing Solid Tumours | 2016/5/1 |

Abbreviation: BsAb, bispecific antibody

**Table S3. Baseline Characteristics of 272 bsAbs clinical trials**

| **Characteristic** | **clinical trials n（%）** | | ***P* value** |
| --- | --- | --- | --- |
|  | **International**  **(excluding China)**  193（71%） | **China-initiated /-involved**  79（29%） |  |
| **Tumor**  Solid Tumor  Hematologic tumor  Both | 112（58）  80（41)  1（1） | 72（91）  5（6）  2（3） | < 0.001* |
| **Center**  single-center  multi-center | 29（15）  164（85） | 43（54）  36（46） | < 0.001* |
| **Solid Tumor**  Lung cancer  Breast Cancer  Colorectal Cancer  Melanoma  Head and Neck cancer  Ovarian Cancer  Prostate cancer  Pancreatic Cancer  Hepatocellular Carcinoma  Renal Cell Carcinoma  Urothelial Cancer  Fallopian Tube cancer  Peritoneal Neoplasms  Cervical Cancer  Sarcoma  Thyroid cancer  Brain and CNS Tumors  Gastric Cancer  Esophageal/GEJ Cancer  Bladder cancer  Biliary Malignant Tumor (BMT)  Nasopharyngeal carcinoma  Thymic Cancer | 26（14）  21（11）  15（8）  14（7）  9（5）  9（5）  8（4）  8（4）  7（4）  6（3）  6（3）  6（3）  5（2）  4（2）  3（2）  3（2）  2（1）  13（6）  9（5）  5（2）  7（4）  3（2）  1（1） | 10（8）  19（16）  7（6）  6（5）  3（3）  2（2）  0（0）  0（0）  3（3）  2（2）  2（2）  0（0）  0（0）  3（3）  0（0）  0（0）  0（0）  18（14）  16（14）  10（8）  8（7）  6（5）  2（2） |  |
| **Mechanism of action**  T-cell redirection  Dual checkpoint blockade  Dual signaling inhibitions  Co-localized blockage  Biparatopic bsAbs  Tumor-targeted immunomodulators  Unclassified | 119（61）  14（7）  37（19）  6（3）  15（8）  1（1）  1（1） | 10（12）  33（42）  2（3）  15（19）  15（19）  4（5）  0（0） | < 0.001* |
| **Time**  Before 2018  After 2018(including 2018) | 85（44）  108（56） | 4（5）  75（95） | < 0.001* |
| **Status**  Recruiting  Active, not recruiting  Completed  Not yet recruiting  Terminated/Suspended/Unknown | 109（59）  15（8）  40（21）  12（6）  12（6） | 56（71）  4（5）  0（0）  17（22）  2（2） | < 0.001* |
| **Enrollment**  Recruiting  Active, not recruiting  Completed  Not yet recruiting  Terminated/Suspended/Unknown | 17891（75）  1133（5）  2106（9）  2180（9）  586（2） | 6306（68）  730（8）  0（0）  2181（24）  21（0） | < 0.001* |
| **Phase**  I  I/II  II  II/III  III | 125（64）  42（22）  23（12）  1（1）  2（1） | 36（46）  12（15）  28（35）  1（1）  2（3） | < 0.001* |

**Abbreviation:** CNS, nervous system tumors; GEJ, Gastroesophageal Junction; BMT, Biliary Malignant Tumor.

**Table S4. Detailed data of accumulative trial locations worldwide corresponding to the Figure S1A**

| **Country/Region** | **Accumulative Trial Locations** |
| --- | --- |
| Argentina | 1 |
| Australia | 37 |
| Austria | 9 |
| Belgium | 21 |
| Brazil | 2 |
| Bulgaria | 2 |
| Canada | 30 |
| Chile | 1 |
| Czechia | 6 |
| China | 98 |
| Denmark | 15 |
| Finland | 1 |
| France | 53 |
| Germany | 54 |
| Hungary | 2 |
| India | 1 |
| Israel | 10 |
| Italy | 27 |
| Japan | 16 |
| Korea | 26 |
| Lebanon | 1 |
| Malaysia | 1 |
| Mexico | 2 |
| Netherlands | 27 |
| New Zealand | 1 |
| Norway | 1 |
| Poland | 16 |
| Portugal | 5 |
| Puerto Rico | 1 |
| Romania | 2 |
| Russia | 7 |
| Saudi Arabia | 1 |
| Serbia | 1 |
| Singapore | 6 |
| Spain | 60 |
| Sweden | 3 |
| Switzerland | 4 |
| Thailand | 2 |
| Turkey | 3 |
| Ukraine | 3 |
| United Kingdom | 33 |
| United States | 145 |

**Table S5. Detailed data of accumulative trial locations in China corresponding to the Figure S1B**

| **City of China** | **Accumulative Trial Locations** |
| --- | --- |
| Anhui | 6 |
| Beijing | 35 |
| Chongqing | 8 |
| Fujian | 10 |
| Gansu | 1 |
| Guangdong | 28 |
| Guangxi | 3 |
| Hebei | 2 |
| Heilongjiang | 10 |
| Henan | 21 |
| Hong Kong | 7 |
| Hubei | 10 |
| Hunan | 13 |
| Inner Mongolia | 1 |
| Jiangsu | 16 |
| Jiangxi | 7 |
| Jilin | 8 |
| Liaoning | 8 |
| Shaanxi | 5 |
| Shandong | 18 |
| Shanghai | 17 |
| Shanxi | 1 |
| Sichuan | 9 |
| Taiwan | 19 |
| Taiwan | 1 |
| Tianjin | 4 |
| Xinjiang | 1 |
| Yunnan | 2 |
| Zhejiang | 17 |

**Table S6. Mechanism of action of bsAbs**

| **Mechanism of action** | **BsAb** | |
| --- | --- | --- |
|  | **International (excluding China)** | **China-initiated /-involve** |
| T-cell redirection | REGN-5678  ,MP0310 ,GEN-1042 ,AGEN-1423,IMCgp100,OXS-1550,AFM-13 ,REGN-1979 ,IMC-C103C ,IMCnyeso ,RG-7828 ,OXS-3550,GEN-3013 ,MCLA-117 ,MGD-006 ,MGD-007 ,REGN-4018 ,AMG-701,AMG-160 ,AMG-330,AMG-424 ,AMG-427 ,AMG-562 ,AMG-596 ,AMG-673 ,AMG-757 ,AMV-564,APVO-436 ,AMG-420 ,RG-6026,EM-901,ERY-974,GBR-1342 ,GEM-333,GEM-3PSCA,IGM-2323 ,JNJ-67571244,JNJ-63709178,JNJ-64007957,JNJ-63898081,MGD-009 ,AMG-212,PF-06671008 ,PF-06863135,REGN-5458 ,RG-6194,TNB-383B ,XmAb-13676,XmAb-14045,XmAb-18087,HPN-424 ,JNJ-64407564 ,RG-6160,SAR-440234 ,AFM-11 ,AFM-24 ,CCW-702 ,HPN-217 ,BI-905711 ,MT110,Hu3F8,MDX447,CDX-527,rM28,removab, MGD006,FS120,REGN5459,REGN7075,GEN1044 ,Catumaxomab,FBTA05,CC-1,ES414,MT103,JNJ-75348780,ABBV-428 | HX-009, IBI-322, A-319, M701, M802, GBR-1302 |
| Dual checkpoint blockade | FS-118, LY-3434172, MGD-013, MGD-019, RG-7769, XmAb-20717, XmAb-22841, RO7247669, LY3415244, MEDI-5752 | KN-046, AK-104, EMB-02, IBI318, SI-B003 |
| Dual signaling inhibitions | ABT-165 ,MP0250,ABL-001,RG-7221 ,BI-836880 ,OMP-305B83 ,MCLA-128 ,PRS-343 ,MCLA-158 ,CodeC2624,LY3164530,MM-111,Amivantamab，EMB-01, JNJ-61186372 | SI-B001 |
| Co-localized blockage | GEN-1046, XmAb-23104, ADC-1015, MCLA-145, BCA101, ES-101 | ES-101, M7824, SHR-1701 |
| Biparatopic bsAbs | BI-905677, MP0274, RG-7802, ZW-49 | KN-026, MBS-301, ZW-25 |
| Tumor-targeted immunomodulators | NI-1701 | IBI-315, IMM0306, AK112 |

**Abbreviation: BsAb, bispecific antibody**
